# Urban visual intelligence: Uncovering hidden city profiles with street view images

**DOI:** 10.1073/pnas.2220417120

**Published:** 2023-06-26

**Authors:** Zhuangyuan Fan, Fan Zhang, Becky P. Y. Loo, Carlo Ratti

**Affiliations:** ^a^Department of Geography, The University of Hong Kong, Hong Kong, China; ^b^Department of Civil and Environmental Engineering, The Hong Kong University of Science and Technology, Hong Kong, China; ^c^School of Geography and Environment, Jiangxi Normal University, Nanchang, 330022, China; ^d^Senseable City Lab, Department of Urban Studies and Planning, Massachusetts Institute of Technology, Cambridge, MA 02139

**Keywords:** urban studies, socioeconomic status, built environment, computer vision, sustainable development goals

## Abstract

A longstanding line of research in urban studies explores how cities can be understood through their appearance. However, what remains unclear is to what extent urban dwellers’ everyday life can be explained by the visual clues of the urban environment. In this paper, we address this question by applying a computer vision model to 27 million street view images across 80 counties in the United States. Then, we use the spatial distribution of notable urban features identified through the street view images, such as street furniture, sidewalks, building façades, and vegetation, to predict the socioeconomic profiles of their immediate neighborhood. Our results show that these urban features alone can account for up to 83% of the variance in people’s travel behavior, 62% in poverty status, 64% in crime, and 68% in health behaviors. The results outperform models based on points of interest (POI), population, and other demographic data alone. Moreover, incorporating urban features captured from street view images can improve the explanatory power of these other methods by 5% to 25%. We propose “urban visual intelligence” as a process to uncover hidden city profiles, infer, and synthesize urban information with computer vision and street view images. This study serves as a foundation for future urban research interested in this process and understanding the role of visual aspects of the city.

## Significance

We demonstrate that urban features extracted from street view images through a computer vision model can effectively estimate the hidden neighborhood socioeconomic status, such as travel behaviors, poverty status, health outcomes and behaviors, and crime. Specifically, models using street view features alone can estimate up to 83% of the variance in vehicle miles traveled, 64% in violent crime occurrences, and 68% in the population lacking physical activities. These results outperform models using other commonly adopted data such as points of interest, population, and demographics. With the increasing availability of street view data and readily available computer vision algorithms, this approach could help estimate urban phenomena that concern sustainable development goals at a finer spatial and temporal resolution.

An in-depth study of the urban environment is vital for knowing cities and the lives within (1–4). The urban environment is a complex system that manifests itself through many measurable patterns, including land use diversity, building density, street network connectivity, presence of greenery, and food and retail business. Leveraging these measures, researchers have broadly established the connection between the urban environment and the urban dwellers’ daily life. For instance, restaurant density and ratings are shown as effective predictors of daytime population, employment, and age (5). The spatial homogeneity of road networks implies cities’ GDP and population growth (6). Accessibility to destinations is strongly associated with the intensity of travel (7, 8). The size of housing and bare agricultural land can be used to infer household poverty levels (9). Access to parks is a consistent predictor of urban health (10–12).

While much of the existing work as such has taken land use and urban function–based measures as key variables to estimate neighborhoods’ socioeconomic status (13–16), we have yet to realize that all urban functions have their visual counterparts. From the early 18th-century architecture theory of “architecture parlante” (17) to more recent works such as Kevin Lynch’s “The Image of the City” (18), there is a consensus that cities can be understood through their appearance. Researchers have extensively tested the impact of the visual attributes on crimes (19, 20), travel behavior (21–23), and health behaviors (24, 25), particularly along street sidewalks. In parallel, planning practices have gradually incorporated zoning codes for building façades, street sidewalks, and street trees to guide the city’s overall appearance. Yet, with all these efforts, an important question remains unanswered: “To what extent is the appearance of cities connected to the multiple aspects of neighborhood socioeconomic status?”

With the advent of high-performance computational methods and ubiquitous street view images, researchers now have new tools to address this research question. Recent literature has applied computer vision models to street view images and estimated income level, voting preference, health outcomes, housing prices, and perception of safety (1, 26–30). Our work shares related interests with these studies but differs by answering two specific questions: 1) local governments across different contexts have spent a lot of efforts in data collection to get a full spectrum of urban lives. Among all these socioeconomic profiles, which ones can be better estimated from objective characteristics of street view images? 2) How well can we infer these socioeconomic profiles from images compared to commonly used function-based measures? To answer these two specific questions, we collected 27 million images from Google Street View (GSV) across 80 counties in seven major metropolitan areas in the United States. By applying a deep learning–based computer vision algorithm (31, 32), we extracted a series of urban features from these images, including trees, sidewalks, cars, building façades, etc. By analyzing the distribution of these street view features in cities, we predicted four aspects of the cities at the neighborhood level: poverty, health, crime, and transport. The focus of these four topics is motivated by the aforementioned literature, which highlights the association between the built environment and these four aspects of socioeconomic status. Moreover, these topics also echo the United Nations’ Sustainable Development Goals (SDGs) 1, 3, 11, and 13 (see *SI Appendix*, Note 1. A Table S1 for details).

With this approach, we show the potential of using computer vision algorithms to estimate poverty, violent crimes, health behaviors, and travel mode preferences through publicly available street view image data. However, the explanatory power of street view images across these targeted variables varies. Travel mode preferences, for instance, are best estimated across all study sites (with an *R*^2^ of 83%), whereas health outcomes such as cancer and mental health occurrence are less explainable through image data alone (with the best *R*^2^ of 48% and 62%, respectively). Notably, in most cases, the model fit (see *SI Appendix*, Tables S8 and S9 for model performance evaluation) using street view images outperforms that of using points of interest (POI) distribution, which has been commonly used as a proxy for urban function. Furthermore, by combining publicly available survey data on race, age, and population density with street view characteristics, we demonstrate that the street view images can still add 5% to 25% additional model fit *R*^2^.

Our approach and results provide empirical evidence for the urban planning theory on the appearance of the city and its importance in citizens’ life. We quantify the strong connection between street view features and many equally important aspects of urban life. This study serves as a preliminary step to the future study of “urban visual intelligence”: using image data to synthesize and infer information of the city and support timely policy intervention.

## Results

**Fig. 1.**
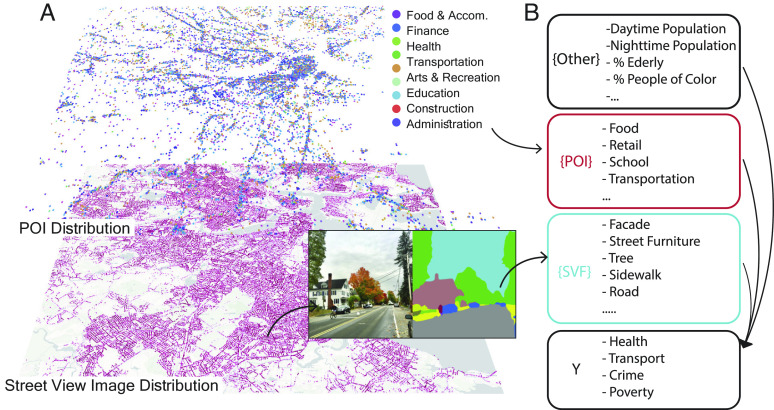
Data and Methodology. (*A*) Schematic of the feature extraction procedure: The study collected GSV and POI data along the street networks in seven selected metropolitan areas. For each sampled GSV, we employed an image segmentation model (31, 32) to extract pixel-level labels of the image, enabling the construction of SVFs (see *SI Appendix*, Fig. S1 for enlarged example. *SI Appendix*, Table S3 for SVF data summary). (*B*) Using features constructed from POI, SVFs, and other demographic variables, we built models to estimate a series of neighborhood socioeconomic variables (*Y*) and compared the model results to assess the estimation power of SVF. These selected *Y*s were chosen based on four main aspects of city life: health, transport, crime, and poverty.

For this study, we collected Google Street View images from seven US metropolitan areas across 9 states (Table 1). The selected metropolitan areas vary in population size and geographical contexts. Fig. 1 shows the overall data and experiment structure.

**Table 1.** Description of each of the metropolitan areas considered and some statistics about our dataset

Metropolitan areas	#POI	#GSV panorama	#Census tracts	#Census block groups	Population	Income per capita
Miami–Fort Lauderdale–Pompano Beach (Miami)	114 K	2.73 M	1,205	3,372	6.02 M	$32,522
Los Angeles–Long Beach–Anaheim (Los Angeles)	245 K	2.03 M	2,767	7,349	12.03 M	$35,916
Chicago–Naperville–Elgin (Chicago)	128 K	7.01 M	1,926	5,541	8.01 M	$38,158
Philadelphia–Camden–Wilmington (Philadelphia)	90 K	3.40 M	1,423	4,058	5.72 M	$39,091
New York–Newark–Jersey City (New York)	283 K	6.89 M	4,446	13,338	18.35 M	$43,409
Boston–Cambridge–Newton (Boston)	71 K	2.54 M	944	3,208	4.52 M	$47,605
San Francisco–Oakland–Berkeley (San Francisco)	77 K	2.39 M	796	2,256	3.52 M	$55,252

We use short names to represent the metropolitan areas in the manuscript. A total of 9 states are included in the study: Massachusetts, New York, New Jersey, Pennsylvania, Delaware, Maryland, Illinois, California, Florida. # stands for count.

We extracted street view features (SVFs) by applying a computer vision algorithm to 27 million GSV images. As described in ref. 31, this algorithm assigns each pixel of an image to a specific semantic category. Out of all categories, we focus on the ones that capture relevant features of the outdoor environment and combine them to create a list of variables: street furniture, sidewalk, facade, window and opening, road, sky, grass, shrubs, trees, people, bikes, and vehicles (further details about the method can be found in *SI Appendix*, Note 1.B). These variables measure the share of pixels of each category relative to the total pixels of the entire image. Similar methods were used to create greenery view index (33, 34), estimate sky opening index(35), measure urban changes (2), and detect abandoned houses (36) in recent studies.

### Using SVF to Estimate Health, Crime, Transport, and Poverty.

We use SVF to estimate health, crime, transport, and poverty statistics. The street view variables were aggregated to different spatial resolutions census tract (CT) and census block group (CBG) provided by the 2010 US Census Bureau. For each spatial unit, we also compute the average value of SVF from immediate neighbors to account for the spatial spillover effect. The variables estimate 18 parameters within the four categories of health, crime, transport, and poverty. For example, we describe poverty by the percentage of the population for whom poverty status is determined as below the 100% regional poverty line and below the 200% poverty line separately. We describe transport using a wide range of travel behavior variables, including vehicle miles traveled (VMT), and the percentage of the population commuting by public transit, car, walking, biking, etc. We then partition the dataset into the training and test sets. We randomly sample 80% of the spatial units for each metropolitan area as the training set and assign the remaining 20% to the test set. We apply a least absolute shrinkage and selection operator (LASSO) regression to each metropolitan area and a spatial resolution with 5-fold cross-validations on the training set (see *Materials and Methods* for details). We report model evaluation results on the test set in this work.

**Fig. 2.**
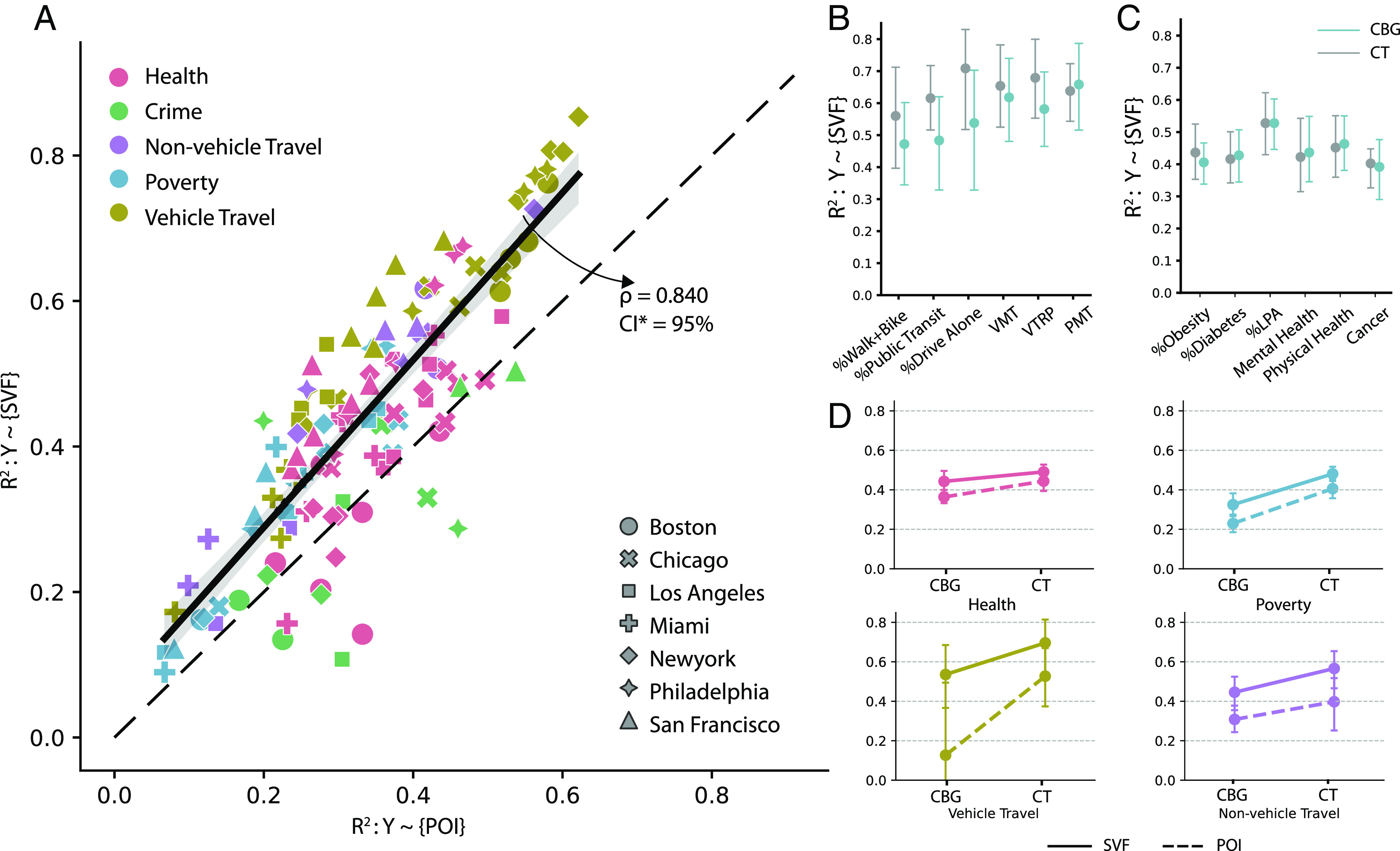
Using SVF to estimate health, crime, transport, and poverty. (*A*) A scatter plot of the model fit *R*^2^ comparison. Models using SVF alone generally outperform models with POI features in estimating most variables (CBG level correlation *ρ* = 0.840; *P*-value < 0.001. The shading indicates 95% CI). We plot the 45-degree line as a reference. The results shown are average test results after repeating the model ten times with random seeding (see *SI Appendix*, Fig. S10 for results at the CT level). (*B*) The *R*^2^ of using SVF to estimate transport-related variables. SVF can account for up to 87% and 85% of the model variance for CT and CBG level estimation, respectively. (*C*) The *R*^2^ of using SVF to estimate health-related variables. The SVF can account for up to 68% and 65% of the variance in the CT and CBG level estimation, respectively (see *SI Appendix*, Figs. S8 and S9 and Tables S8 and S9 for full results on other dependent variables). (*D*) Comparing the model fit *R*^2^ between using SVF and POI features at both CBG and CT levels. In general, the CT-level models show a better fit than the CBG-level models (mean difference 6.8%, *P*-value < 0.001).

Overall, the SVF estimates the transport-related urban parameters at best: the model fit *R*^2^ on the test set reaches 87% when estimating the percentage of the population commuting by driving alone and 82% when estimating the percentage of the population commuting by public transit. The model *R*^2^ also reaches 68% on the percentage of people lacking physical activities (LPA) (Fig. 2*B*), 64% on violent crimes, and 62% on the percentage of individuals with income below 200% poverty line. In contrast, we found that the model performs at relatively lower *R*^2^ on the percentage of the adult population with cancer, diabetes, and mental health issues (CT-level *R*^2^ best estimated at 48.1%, 59.3%, and 62%, respectively, on the test set, Fig. 2*C*, *SI Appendix*, Table S9). These results are generally consistent across metropolitan areas (*SI Appendix*, Figs. S8 & S9).

### SVF Outperforms POI in Socioeconomic Profile Estimation.

To justify the prediction power of SVF, we repeat the models using POIs, one of the most commonly used features in data-driven urban studies, as a comparison. Our POI data are from the Safegraph core data (2019), National Park and Recreation, and the Transit-oriented Development Database (See *SI Appendix*, Table S5 for sources of data). The Safegraph core data contain each POI’s category (by NAISC code), address, latitude, and longitude. Similarly to the aggregation of SVF, we aggregate the POI features by spatially joining them to the spatial units. Then, we count the number of POIs by each category in each spatial unit. The category includes retail, food and accommodations, education, transportation, public administration, manufacturing, and construction (see *Materials and Methods* for details). For each spatial unit, we also compute the park accessibility and rail transit accessibility. The park accessibility is described by the area of park space within an 800-m buffer, and the rail transit accessibility is defined by the distance to the closest rapid-rail transit station. In total, we include 39 features in the POI model.

Fig. 2*A* compares the model performance between the SVF and POI models at the CBG level. Our experiments show that models using SVF outperform POI models in 110 out of 124 cases at CBG level and 97 out of 124 cases at CT level (*SI Appendix*, Fig. S10). Since the *R*^2^ of the two sets of models share a linear relationship (*ρ* = 0.856, *P* < 10^−30^), one may question whether some of the information in the two datasets overlaps. To address this, we conducted an experiment that included both sets of data in one model and computed the feature group importance. As shown in *SI Appendix*, Fig. S9 (*SI Appendix*, Note 1B), SVF contributes more on average than the POI feature group, particularly in predicting transport-related aggregated behaviors.

To further justify the robustness of our results, we compare the model results at CBG and CT levels. Fig. 2*D* presents that the model’s *R*^2^ is generally higher at the CT level than at the CBG level. This phenomenon could be attributed to the fact that CTs, being larger in size than CBGs, tend to exhibit lower levels of heterogeneity. As a reference, we also show the model estimation results using POI only and found a similar trend between the CT and CBG level estimation.

### SVF Outperforms Dynamic Population in Socioeconomic Profile Estimation.

One may question whether the distribution of POI and SVF can provide similar information to that of population density. If this is the case, can a similar model performance be achieved by using population-related factors alone? Indeed, our study shows a strong correlation (Pearson *ρ* = 0.71, *P* < 0.001) between the average proportion of building façades and population density. Besides residential population density, recent studies have also analyzed the relationships between daytime population, the number of visitors to each geographical location, crime, transport, and health-related activities (13, 37). Following this thread, we construct a series of models to test how well dynamic population features alone can predict the aspects of cities covered in this study. The dynamic population is described by day-time (visiting) population, population density, night-time (residential) population, and residential density.

**Fig. 3.**
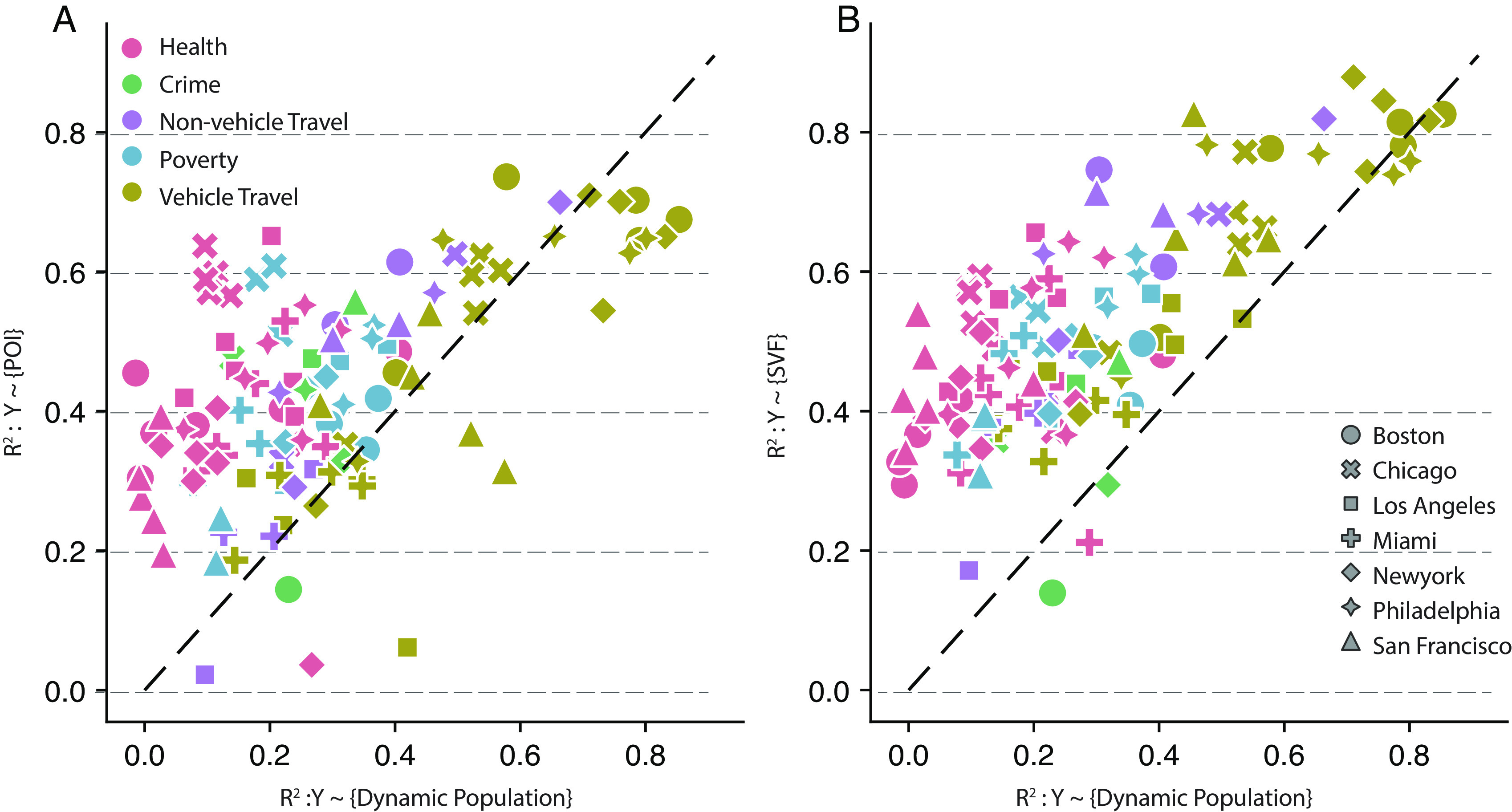
Model fit *R*^2^ comparison among SVF, POI, and dynamic population. (*A*) The *R*^2^ comparison between models using population alone and models using POI features alone. On average, the *R*^2^ of the CT-level model (*Y* ∼ {*POI*}) is about 9.8% higher (*t*-test; *P*-value < 0.005) than the *R*^2^ of model (*Y* ∼ {*DynamicPopulation*}) (*SI Appendix*, Table S9). (*B*) The *R*^2^ comparison between models using population alone and models using SVF alone. On average, the *R*^2^ of model (*Y* ∼ {*SVF*}) is about 22.8% (*t*-test; *P*-value < 0.005) higher than the *R*^2^ of model (*Y* ∼ {*DynamicPopulation*}) (*SI Appendix*, Table S7). We annotate the 45-degree line as a reference. Any dots above the line indicate that the model outperforms the model as a function of population estimation.

Our night-time population data is from ACS 5-y survey (2015 to 2019), and our daytime population data are computed from Safegraph neighborhood visiting pattern data (see *Materials and Methods* for details). Fig. 3 shows that both SVF and POI features are stronger estimators than dynamic population features for most models. However, we observe that population features strongly connect with vehicle travel-related parameters: the model reaches an *R*^2^ of 83% in estimating the VMT in Boston and New York City. In contrast, population features have neglectable prediction power in estimating health-related parameters.

### Beyond Traditional Measures—SVF Offers Additional Information for Estimating Transport, Health, Poverty, and Crime.

It is well-studied in the US context that demographic factors such as age and race are connected with neighborhood well-being. For example, recent studies show that COVID-19 mortality and infection rates are related to racial inequality (38, 39). Aging is also one of the core factors that are related to health outcomes such as cancer and diabetes. People’s travel mode choice could be highly associated with their geographical locations (40). Here, we examine whether the addition of SVF can improve the estimation of transport, health, poverty, and crime, under the scenario when the basic demographic information is collected. The demographic features include residential population, percentage of people of color (non-White population), percentage of people over 65 y old, and distance from each spatial unit to the downtown area (see *Materials and Methods* for variables’ details).

To compare the models, we also evaluate the incremental change in model *R*^2^ by separately incorporating each type of feature and subsequently adding the other type. To begin with, the first set of equations, i) *Y* ∼ {*SVF*}, models all four targeted variables using SVF alone, and ii) *Y* ∼ {*Loc*.+*Pop*.+*Age* + *POC*} models all targeted variables using basic demographic profiles of each spatial unit. The second set of equations, iii) *Y* ∼ {*SVF*}+{*Loc*.+*Pop*.+*Age* + *POC*}, combines all variables in the previous two models. The model *R*^2^ of the first (SVF or demographic alone) and second (combined) sets of equations are then compared to estimate the incremental increase in model performance (see *Materials and Methods* for details).

**Fig. 4.**
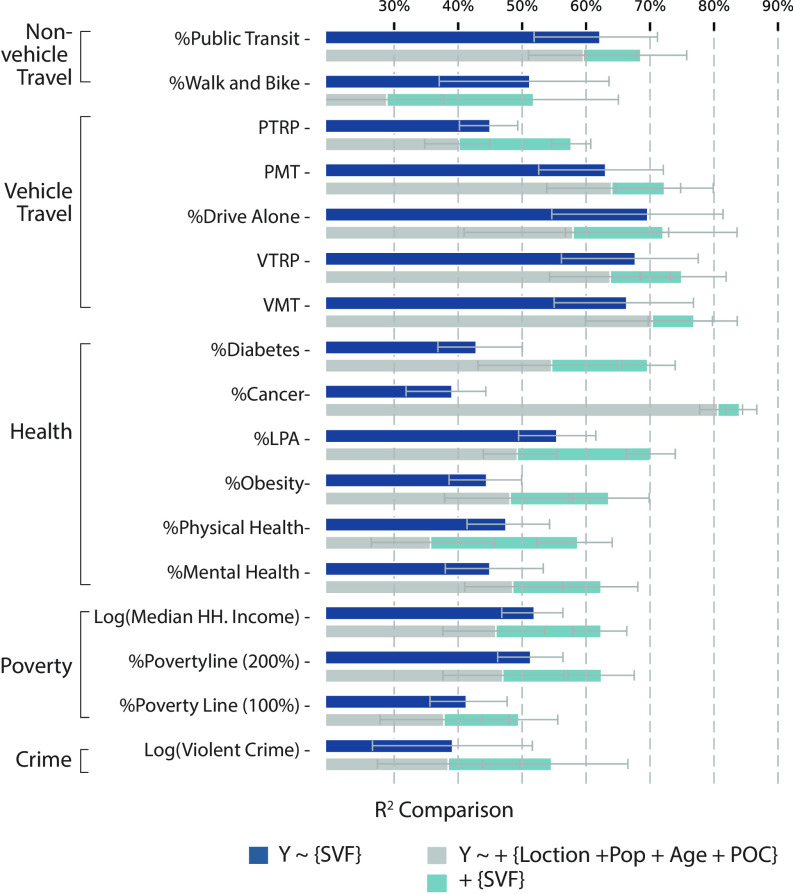
Multivariate analysis of all city life indicators. The dark blue bars represent the *R*^2^ when models only consider the SVF. The gray bars represent the *R*^2^ when models only consider the location, population, age, and people of color. The light blue bar indicates the additional *R*^2^ gained by adding SVF to the previous model. Error bars are presented for each model result. On average, by adding the SVF to the model, we are able to add 5% to 25% to the model *R*^2^. *Age* stands for the percentage of the population over 65. *POC* stands for the percentage of the population from a non-White race group.

Fig. 4 shows two main findings. First, models using SVF outperform demographic features alone or demonstrate comparable prediction power when estimating transport, poverty, and crime. However, regarding health outcomes, SVF outperforms demographic features when estimating physical inactivity (%LPA) and physical health status. On the contrary, for diseases such as cancer and diabetes, demographic features are more robust predictors. These results echo that age and race are strongly connected with common diseases, while SVF has a limited ability to capture the variance. Second, the addition of SVF to the model increases the predictive capacity by 5% to 25% when compared to using demographic features alone. The models that predict people commuting by walking and biking, physical inactivity, physical health issues, and the count of violent crimes have gained the most significant increase. These variables are all related to the intensity of human activities, which is in concert with existing studies that assert the deep connection between human activities and the urban environment (41).

## Discussion

This work makes three primary contributions to understanding urban lives via computer vision tools. First, we quantify the value of street view features (SVFs) in uncovering neighborhood socioeconomic statistics of a wider variety. Empirical urban studies that have leveraged computer vision tools tend to focus only on narrow outcomes such as wealth (3), crime (42), or health (10, 28). The well-being of urban life, however, consists of a much wider range of behavior states, choices, and outcomes. A parallel examination across multiple equally important aspects of urban life has been notably absent. Here, our study shows that SVFs best estimate travel behaviors but are poorest at estimating certain health outcomes such as cancer, diabetes, and mental health issues. This result is consistent across different urban contexts (*SI Appendix*, Figs. S8 and S9). As such diseases are closely related to adult mortality rates, planning officials should pay further attention in detail data collection in these realms. Second, by comparing SVF’s prediction capacity with other commonly used urban measures, we conclude that SVF outperforms data sources such as POIs and dynamic populations. POIs have been widely used as a proxy for urban functions to infer the vitality of cities (5), and the dynamic population is also a core variable in urban growth theories. Our result indicates that the overall “look” of the built environment may contain more information than functions, residential, and visitor activity density. Last, disaggregated and cross-context comparable data are essential for UNhabitat’s SDG monitoring. Considering the spatial–temporal resolution and the global coverage of the street view images, previous studies have proposed to use computer vision tools and street view images to complement the labor-intensive data gathering approach(1, 2). Building on these studies, by comparing the models using SVF with those using age, population, and people of color variables alone, we reveal that the magnitude of information gained by adding SVF to the prediction model varies across different aspects of urban life.

Similar to how perceived facial age can serve as a valid marker of an individual’s health, “the look” of the urban environment is demonstrated here to be highly connected with the well-being of a city. This knowledge is particularly valuable for policy makers and urban planners, who can adopt early interventions to help neighborhoods with potential problems of obesity, violent crimes, or poverty, rather than waiting for prolonged survey results.

This study serves as a baseline approach for future studies in the realm of “urban visual intelligence”. With increasing available computer vision tools and urban data, researchers can further extract semantic meanings from the images and videos of cities. These tools and data allow urban studies to capture large-scale microvariations in cities, synthesize hidden information in cities, and infer future trends. Moreover, planning strategies in practice today are still predominantly around land uses and functions. Research advances in the visual appearance of cities can further influence planning methodologies in the future and bring the “look” of the city into actionable planning strategies.

The results of our study should be interpreted in light of its limitations. We only obtain street view images where Google Street View has coverage, which may have omitted private and very-high-income areas. In addition, other important socioeconomic factors mentioned by SDGs, such as accessibility to healthy food, education outcomes, sanitation, and traffic accidents, also deserve further exploration. The methods in this study can be applied to these targeted variables to reveal the potential and limitations of using SVF to measure cities.

## Materials and Methods

### Study Site.

This study includes seven metropolitan areas in the United States, abbreviated as follows: Miami–Fort Lauderdale–Pompano Beach (Miami), Los Angeles–Long Beach–Anaheim (Los Angeles), Chicago–Naperville–Elgin (Chicago), Philadelphia–Camden–Wilmington (Philadelphia), New York–Newark–Jersey City (New York), Boston–Cambridge–Newton (Boston), and San Francisco–Oakland–Berkeley (San Francisco). Table 1 presents the summary statistics. To ensure statistical reliability, only CTs and CBGs with a minimum of 30 residents are included in the study.

### Street View Features (SVFs) from the Street View Images.

Street view panoramas are downloaded via Google Street View API. We obtain four cutouts of each panorama by specifying the heading and pitch of the camera relative to the street view vehicle. To ensure data quality, we apply several filtering criteria to the obtained street view images: 1) images that were taken either in winter months (January, February, November, and December) or before 2016 are excluded; 2) images that were taken at major highways, as defined by OpenStreetMap’s highway category, are excluded; 3) after the segmentation process, images that contain no pixel of sidewalk were dropped since they are likely taken at places inaccessible to pedestrian or too far from the sidewalk.

After this process, we summarize the number of images by each spatial unit. Only CBGs or CTs with more than 20 street view panoramas are included in the study. On average, each CBG (smallest unit in the study) contains over 700 image samples.

We used a semantic segmentation model trained on the MIT ADE20K scene parsing dataset to classify the pixel in each street view image. The architecture used in this study is ResNet18dilated + PPM_deepsup. The model is available at https://github.com/CSAILVision/semantic-segmentation-pytorch. The original dataset contains 150 categories, from which we select 38 categories and group them into 13 street view features {*SVF*} (see *SI Appendix*, Table S2 for our method of grouping the labels). The following equation is used to calculate the proportion of feature *i* in four images cut collected to create a panoramic view:

[1] $$\begin{array}{c} SVF_{i}=\frac{\sum_{j}^{n=4}Pixel_{j}^{i}}{\sum_{j}^{n=4}Pixel_{j}}\times 100\%, \end{array}$$

where *Pixel*_*j*_^*i*^ is the number of pixels that are classified as feature *i* at direction *j*, and *Pixel*_*j*_ is the total number of pixels of the image cutout at direction *j*. Examples of SVF distribution are shown in *SI Appendix*, Figs. S2 and S3.

### POI Measures.

The POI measures include the aggregated count of different types of POIs, distance from the closest rail transit station, and accessible park areas (within an 800-m buffer) per capita. POI data were extracted from Safegraph core data. Each POI place includes the parameters of latitude, longitude, location name, address, and NACIS code. Based on the two-digit NACIS code, we grouped the POIs into nine categories, namely retail, food and accommodations, arts and recreation, education facilities, financial institutions, transportation facilities, health care centers, construction, and manufacturing (see *SI Appendix*, Table S4 for a summary of POI by category for each metropolitan area). We tallied the total number of POI per category within each spatial unit. To ensure that our results are not dependent on a specific POI data source, we compared our dataset with POI data from Reference USA historical data. The distribution of different types of POI between the two datasets is highly correlated (*ρ* = 0.8971) (*SI Appendix*, Fig. S4).

Distance from the closest rail transit station is calculated as the great-circle distance between the center of each spatial unit to the closest rapid rail station. The accessible park area per capita is calculated as the park area falling within the 800-m buffer of each spatial unit divided by the local population.

### Targeted Neighborhood Socioeconomic Variables.

The dependent variables include four major sources: health indicators from the Center for Disease Control and Protection’s 500 city project, vehicle travel habits from the 2017 National Household Travel Survey, other travel habits from the ACS 2015 to 2019 (5-y) survey, and crime data from each city’s open data website (see *SI Appendix* for details on data aggregation). A summary of statistics of all targeted neighborhood socioeconomic variables used in this study is shown in Table 2.

**Table 2.** Summary statistics of all dependent variables

Topic	Y	count	mean	std	min	max
Crime	Log(Violent Crime)	4,605	2.77	1.11	0.00	6.91
	Log(Theft-related Crime)	4,605	2.96	1.59	0.00	7.91
Nonvehicle travel	%Walk+Bike	13,104	5.19	8.26	0.00	100.00
	%Public transit	13,104	18.68	20.67	0.00	100.00
Vehicle travel	%Drove alone	13,104	62.05	23.09	0.00	100.00
	VMT	12,608	32.11	12.50	4.66	73.70
	VTRP	12,604	4.47	1.34	0.88	7.28
	PTRP	12,628	8.36	1.14	4.67	11.26
	PMT	12,614	50.39	13.67	21.43	98.97
Health	%Obesity	11,450	27.37	6.26	11.90	50.80
	%Diabetes	11,450	10.36	3.56	0.70	44.20
	%LPA	11,450	26.25	8.10	10.50	63.70
	%Mental health	11,450	13.55	3.16	6.50	31.90
	%Physical health	11,450	12.12	3.52	3.30	37.60
	%Cancer	11,450	6.02	1.99	0.60	20.40
Poverty	Log(Median Household Income)	13,061	11.19	0.51	9.13	12.43
	%Poverty %200	13,099	29.15	18.68	0.00	100.00
	%Poverty %100	13,099	13.28	11.14	0.00	100.00

This table summarizes all dependent variables at the CBG level. See *SI Appendix*, Table S6 for the CT-level summary. The distribution of dependent variables on the map is shown in *SI Appendix*, Fig. S6.

### Other Variables.

For the neighborhood daytime population, we select data from June, July, August, November, December, and January in 2019 at CBG level Safegraph weekly pattern to compute the average daily visiting volume (see *SI Appendix*, Table S5 for further details and all additional data sources).

### Predicting Neighborhood Socioeconomic Profiles with SVF.

To estimate neighborhood socioeconomic statistics, we train LASSO regression models using the Python package scikit-learn. To avoid potential overfitting of the model, we split the data into 80% training and 20% test sets. We further split the training set into five randomly sampled folds to conduct cross-validation. This process was repeated ten times using different random seeds to determine the average performance of the test set. The same process was repeated at both CBG and CT levels to ensure that our results were robust (see *SI Appendix*, Note 2.A for model results on different spatial resolutions). In addition, we also include POI and SVF in one set of models and calculate the permutation importance to show the different contributions of SVF and POIs (see *SI Appendix*, Note 2B and Fig. S11 for details).

## Supplementary Material

Appendix 01 (PDF)

## Data Availability

The analysis was conducted using Python. Code to reproduce the main results in the figures from the aggregated data is publicly available on the GitHub repository (https://github.com/brookefzy/urban-visual-intelligence) (43). All study data are included in the article and/or *SI Appendix*.
