# Supplementary material for: Urban visual intelligence: Uncovering hidden city profiles with street view images
Source: Proc Natl Acad Sci U S A. 2023 Jun 26;120(27):e2220417120. doi: 10.1073/pnas.2220417120 (PMC10319000; doi:10.1073/pnas.2220417120)
Supplement: Supplementary file 1 — Appendix 01 (PDF) [file pnas.2220417120.sapp.pdf]

Supplementary Information for

Urban visual intelligence: uncovering hidden city profiles with street view images

Zhuangyuan Fan, Fan Zhang, Becky P.Y. Loo, Carlo Ratti

Corresponding author: Fan Zhang.
E-mail: cefzhang@ust.hk

This PDF file includes:

Supplementary text
Figs. S1 to S11
Tables S1 to S9
SI References

Contents

1 Data Details **3**
- A Choice of neighborhood socioeconomic statistics to predict . . . 3
- B Street view features (SVF) from Google Street View (GSV) images . . . 3
- C Point of interest (POI) data . . . 5
- D Robustness against the choice of POI dataset . . . 7
- E Other Data . . . 7
  - E.1 Data Source . . . 7
  - E.2 Spatial Resolution . . . 7
  - E.3 Neighborhood socioeconomic statistics . . . 8
  - E.4 Day time population . . . 8
  - E.5 Crime . . . 8

2 Model Estimation **9**
- A Robustness test with other spatial resolution . . . 9
  - A.1 Census Block Group Level Result . . . 9
  - A.2 Census Tract Level Result . . . 9
- B Feature Group Importance Comparison between POI and SVF . . . 9
- C Compare the effects of SVF, POI, and Dynamic Population . . . 11

3 Software used **16**

4 Code Availability **16**

List of Figures

- S1 Image segmentation example . . . 4
- S2 Distribution of grasses detected from the GSV . . . 5
- S3 Distribution of grasses detected from the GSV part B . . . 6
- S4 Comparison between the *ReferenceUSA* and *Safegraph* data. . . . 8
- S5 Distribution of the socioeconomic variables to predict-part A . . . 9
- S6 Distribution of the socioeconomic variables to predict-part B . . . 10
- S7 Spatial distribution of average summer visiting volume on a weekday . . . 17
- S8 All census block group (CBG)-level models in detail . . . 18
- S9 All census tract (CT)-level models in detail . . . 19
- S10 Compare the POI models with SVF models at the census track (CT) level. The shading indicates 95% confidence interval. . . 20
- S11 Relative permutation importance of SVF and POI features . . . 21

List of Tables

- S1 SDG and neighborhood variables . . . 3
- S2 Visual features taxonomy combined from the original ADE 20 Labels . . . 4
- S3 Summary Statistics of Visual Features (Percentage of each Visual Feature in an Image) . . . 7
- S4 Summary of POI Count by Metropolitan Area . . . 7
- S5 Data Source . . . 8
- S6 Summary Statistics of All Dependent Variables (CT level) . . . 11
- S7 Compare model fits among SVF, POI, and dynamic population at census tract (CT) and census block group (CBG) levels . . 11
- S8 Full Model Results (CBG level) . . . 12
- S9 Full Model Results (CT level) . . . 14

Supporting Information Text

1. Data Details

A. Choice of neighborhood socioeconomic statistics to predict. This research mainly concerned the SDGs' indicators that suffer from short of timely and disaggregated data. We pick several neighborhood socioeconomic variables closely related to the SDG targets as implied in Table S1.

Table S1. SDG and neighborhood variables

	SDGs	Variables
Goal 1	No Poverty	Median Household Income
		% Individuals with poverty status determined: below 100% poverty line
		% Individuals with poverty status determined: below 200% poverty line
Goal 3	Good health and well-being	Model-based estimate for crude prevalence of diagnosed diabetes among adults aged >=18 years
		Model-based estimate for crude prevalence of no leisure-time physical activity among adults aged >=18 years
		Model-based estimate for crude prevalence of obesity among adults aged >=18 years
		Model-based estimate for crude prevalence of cancer (excluding skin cancer) among adults aged >=18 years
		Model-based estimate for crude prevalence of physical health not good for >=14 days among adults aged >=18 years
Goal 11	Make cities inclusive, safe, resilient and sustainable	Violent crime occurance per spatial unit
		Violent theft-related crime occurance per spatial unit
Goal 13	Climate change	% Population (>16) commute by walking
		% Population (>16) commute by public transit
		% Population (>16) commute by driving alone
		% Population (>16) commute by biking
		Estimated vehicle miles traveled on a working weekday
		Estimated personal miles traveled on a working weekday
		Estimated vehicle trips traveled on a working weekday
		Estimated personal trips traveled on a working weekday

B. Street view features (SVF) from Google Street View (GSV) images. We keep 27 million Google Street View images for the seven metropolitan areas. Examples of visual feature aggregation to maps are shown in Figure S2 & S3.

We extracted urban visual features by applying an image semantic segmentation algorithm[1,2] to 27 million 360 panorama images of streetscapes sampled across 7 U.S. metropolitan areas. The panorama images were obtained through the GSV application programming interface. Each panorama was associated with a unique identifier, latitude, longitude, and month and year of the image captured. Only images taken between 2016 and 2019 and March to October were included in the study to minimize seasonal effects. In addition, images that were taken along highways were excluded from the study to focus on pedestrian-accessible spaces.

The original segmentation algorithm[1] was trained with the MIT ADE20K scene parsing dataset. The model assigns each pixel of an image to a given category out of 150 categories in total. Out of all categories, we focus on 38 categories that capture relevant features of the outdoor environment and combine them to create a list of 13 variables: street furniture, sidewalk, facade, window & opening, road, sky, grass and shrubs, trees, people, bike, vehicles. These variables measure the share of pixels of each category relative to the total pixels of the entire image. Specifically, the street furniture category includes pixels that are classified as chairs, benches, streetlights, and awnings. The selection of these features was inspired by previous works that articulate the connection between street design and street activities, such as the importance of street furnishing in urban exploration[3,4], the impact of shading on pedestrian route selection[5], the relationship between "eyes on the streets" and urban crimes[6,7], etc.

Examples of visual features extracted from the street view images are shown in Fig.S1.

A summary of all visual features is included in Table S3.

Table S2. Visual features taxonomy combined from the original ADE 20 Labels

No.	Visual Features	Original Category in ADE 20 Dataset
1	Person	person;individual
2	Bike	bicycle;bike;wheel;cycle
		minibike;motorbike
3	Heavy Vehicle	truck;motortruck
4	Light Vehicel	van
		car;auto
		bus;autobus;
5	Façade	wall
		house
		building;edifice
		skyscraper
		hovel;hut;hutch;shack;shanty
6	Window & Opening	windowpane;window
		door;double;door
7	Road	road;route
8	Sidewalk	sidewalk;pavement
9	Street Furniture	streetlight;street;lamp
		bench
		chair
		seat
		awning;sunshade;sunblind
		booth;cubicle;stall;kiosk
		signboard;sign
10	Greenery - Tree	tree
		palm;palm;tree
11	Greenery - Grass & Shrubs	grass
		plant;flora;plant;life
		flower
12	Sky	sky
13	Nature	hill
		lake
		waterfall
		mountain
		water
		river
		sea
		rock

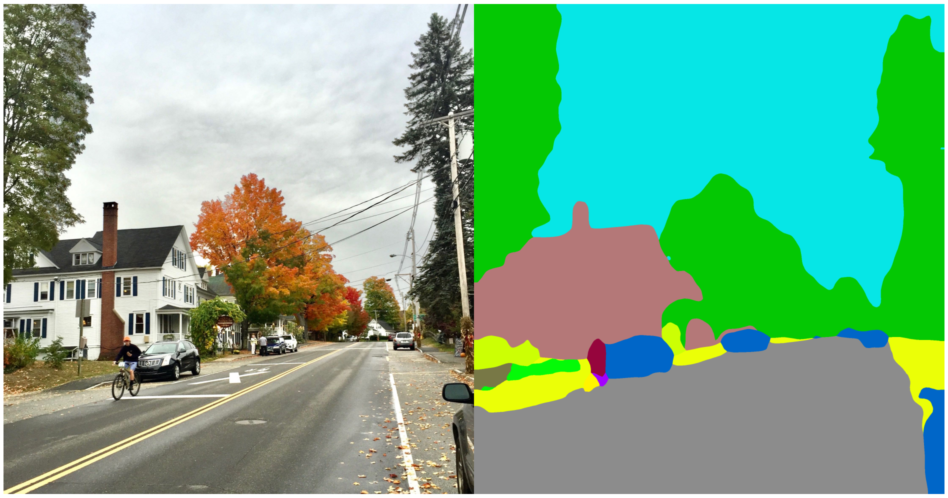

Fig. S1. Image segmentation example

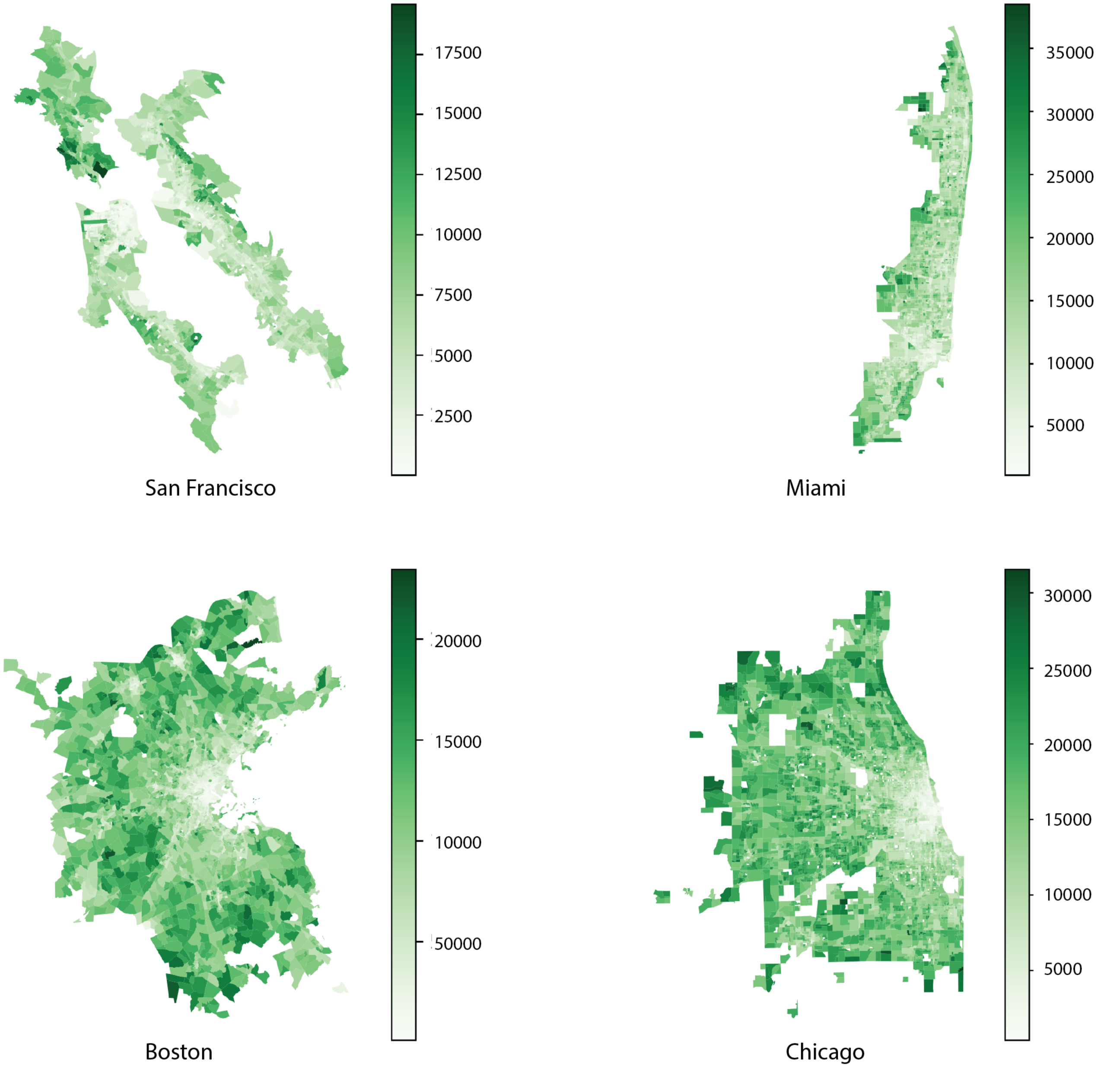

Fig. S2. Distribution of grasses detected from the GSV

C. Point of interest (POI) data.

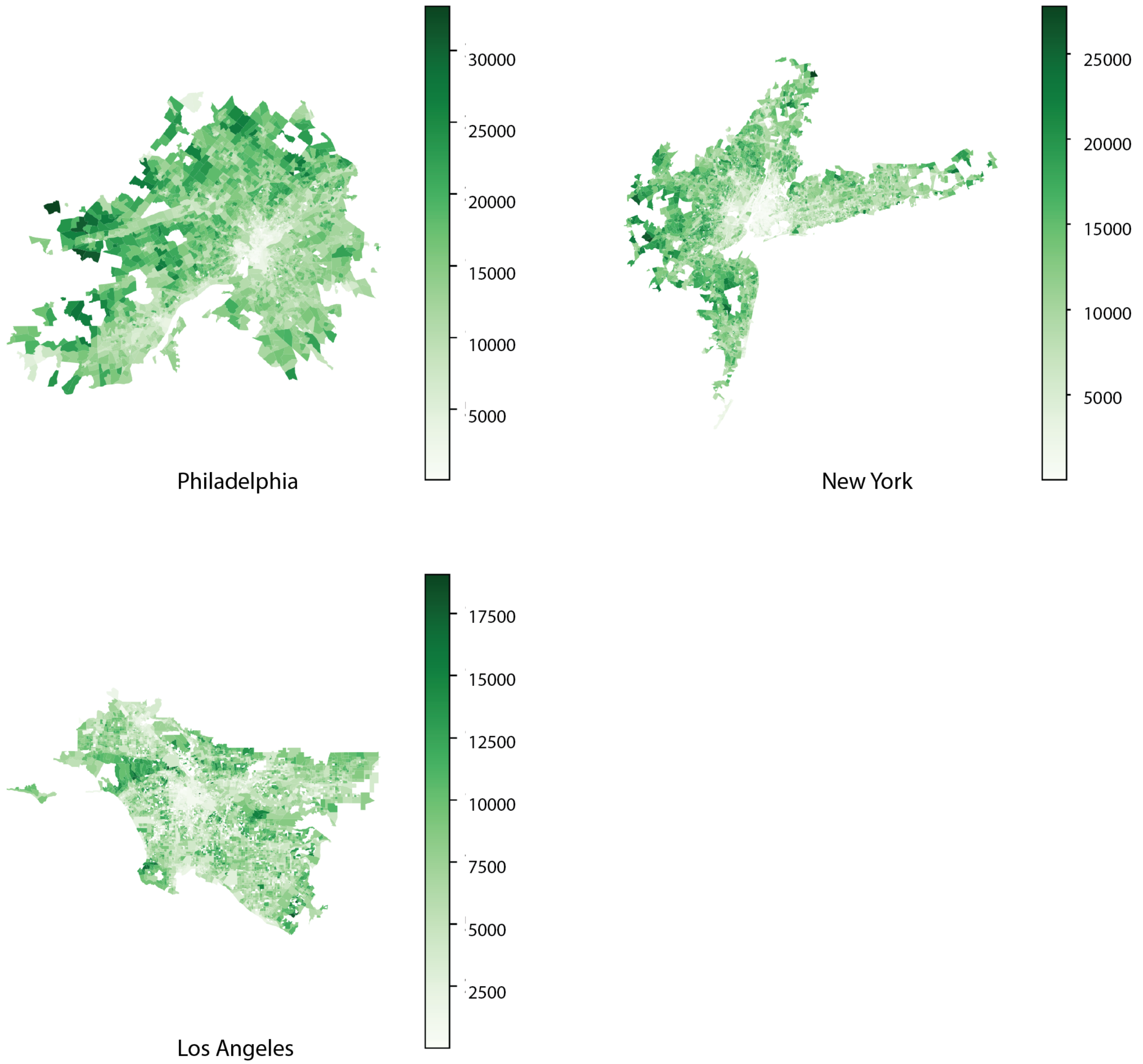

Fig. S3. Distribution of grasses detected from the GSV part B

The functional features came from three major sources. Our first source is a collection of 1.1 million Points of Interest (POIs) across all the study areas downloaded from the Safegraph data store[8]. Based on the first two digits of the NASIC code, we selected and grouped all POIs into the following categories: food and accommodations, retails, finance, construction, manufacturing, transportation, education, arts and recreation, and health. Each POI contains a unique id, location name, latitude, and longitude. Then we aggregate the available number of each type of POIs to its associated census block group. Our second data source is the park area across all metropolitan areas obtained from the National Park and Recreation database[9]. We calculate the available park area within a one-kilometer buffer of each census block group as the available park area. The last data source captures the distance from each census block group to its closest railway station. The location, latitude, longitude, and name of each station are downloaded from the National TOD database[10]. The distribution of POI types by metropolitan area is listed in Table S4.

Table S3. Summary Statistics of Visual Features (Percentage of each Visual Feature in an Image)

	Observation	mean	std	min	max
Sky	32529	0.257	0.078	0.009	0.491
Road	32529	0.256	0.039	0.049	0.416
Planting	32529	0.251	0.119	0.001	0.751
Facade	32529	0.125	0.096	0.000	0.601
Sidewalk	32529	0.047	0.015	0.004	0.188
Vehicle	32529	0.040	0.031	0.000	0.189
Ground	32529	0.012	0.013	0.000	0.197
Street Furn.	32529	0.002	0.001	0.000	0.018
Nature	32529	0.001	0.003	0.000	0.168
Window	32529	0.001	0.001	0.000	0.018
Recreation	32529	0.001	0.002	0.000	0.097
Landmark	32529	0.000	0.001	0.000	0.036
Person	32529	0.000	0.001	0.000	0.024
Bike	32529	0.000	0.000	0.000	0.008

Table S4. Summary of POI Count by Metropolitan Area

POI Category	Boston	Chicago	Houston	Los Angeles	Miami	New York	Philadelphia	San Francisco
Accommodation	11539	20729	14569	37016	15198	53844	13905	12719
Administration	531	1013	1174	2669	1488	1627	658	868
Arts	5562	8637	3848	11483	5356	14915	5013	5117
Construction	64	153	206	533	308	236	95	168
Education	2501	4647	2799	7553	3182	10422	3326	2522
Finance	3902	9117	6474	13507	7495	13523	5547	3401
Health	11494	23484	14443	41965	18726	54736	17926	15364
Information	895	2064	1522	3150	1628	3981	1165	922
Manufacturing	662	1194	807	2548	1123	2810	811	838
Professional	1047	2058	1727	4717	2073	4115	1344	1206
Public Administration	629	888	453	1334	608	1754	650	623
Real Estate	1381	2695	2723	5118	6431	4702	1829	1460
Retail	16869	28096	22543	59122	27189	64680	20636	16277
Transportation	336	632	402	1219	655	1214	353	497
Utitlies	134	52	28	146	16	241	90	33
Warehouse	488	735	492	1572	664	1822	633	581
Wholesale	343	502	563	1500	673	875	431	473
Total	58377	106696	74773	195152	92813	235497	74412	63069

D. Robustness against the choice of POI dataset. To make sure that our result does not subject to the selection of the POI dataset, we gather data from "ReferenceUSA Business historical Data"[11], which is a record do f companies across the US. The dataset is provided annually by Infogroup's U.S. Business Database (We use the 2019 version). The data, similar to the Safegraph data, contains the company name, mailing address, SIC and NAICS codes, employee size, sales volume, latitude, and longitude for each company. We first compare our dataset by each census tract with the ReferenceUSA data. Fig S4 shows the comparison. The summary of the two data at the CT level is highly correlated ($\rho = 0.897, p < 0.0001$).

E. Other Data.

E.1. Data Source. All raw data sources are listed in Table S5.

E.2. Spatial Resolution. We mainly test census block group and census tract level estimation in this study. We only keep the spatial units with more than 50% area covered by land. The original shapefile was called using the US Census API 2019 version of the 2010 census data. Note that census shapefiles change every ten years, thus we limit all our studies until 2019 to avoid inconsistency.

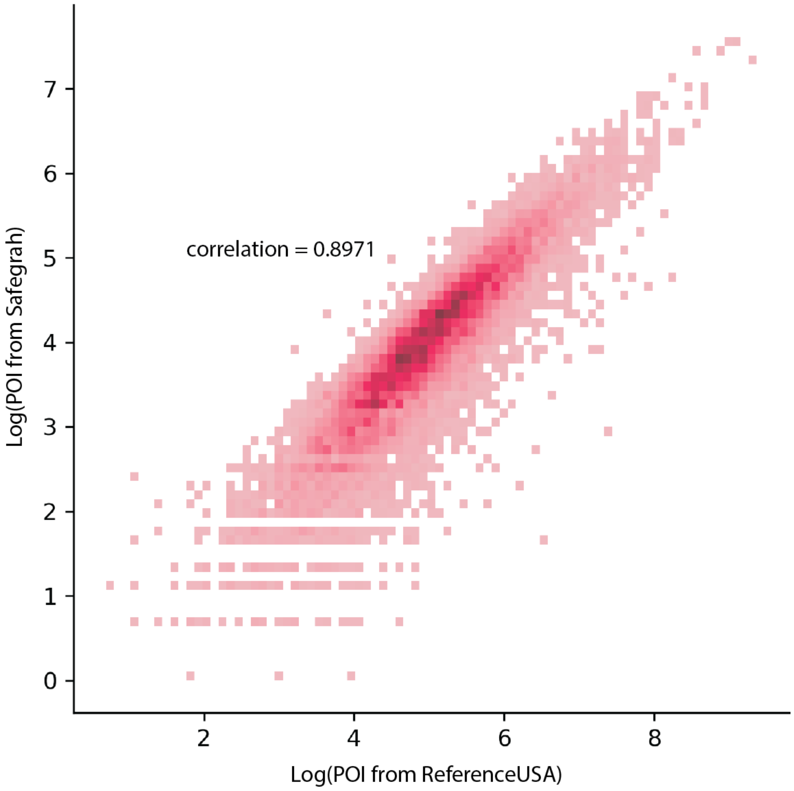

Fig. S4. Comparison between the *ReferenceUSA* and *Safegraph* data.

Table S5. Data Source

Variable Groups	Provider Name	Data Source
1. Health Outcomes	Local Data for Better Health	https://www.cdc.gov/places/
2. Travel Behaviors	2017 National Household Travel Survey	https://www.bts.gov/
3. Online Reviews	Yelp	https://www.yelp.com
4. Commute by Bike and Walk	2015 -2019 American Community Survey	
5. Station Location	Transit-Oriented-Development (TOD) database	https://toddata.cnt.org/
6. POI venues	Safegraph Core Place	https://catalog.safegraph.io/
7. POI venues	Reference USA Historical Dataset	MIT Libraries Dataverse
8. Park Area	National Park and Recreation	https://public-nps.opendata.arcgis.com/
9. Census Block and Census Tract Geometry	United States Census Bureau	United States Census Bureau Geography Files

E.3. Neighborhood socioeconomic statistics. A summary of statistics of all dependent variables to predict is listed in Table S6. Here census tracts and census block groups with fewer than 5 POI or 20 GSV samples are excluded from the summary.

E.4. Day time population. The daytime population data is obtained from Safegraph Census Block Group neighbourhood visiting pattern data[12]. An example of the distribution summer visiting pattern is shown in Fig. S7.

E.5. Crime. The crime data were downloaded from each city's own website. Crime reports are downloaded from:

- New York City Crime Incident[13],
- Boston Crime Incident[14],
- Philadelphia Crime Incident[15],
- Cambridge Crime Incident[16],
- Los Angeles Crime Incident[17],
- San Francisco Crime Incident[18],
- Chicago Crime Incident[19],

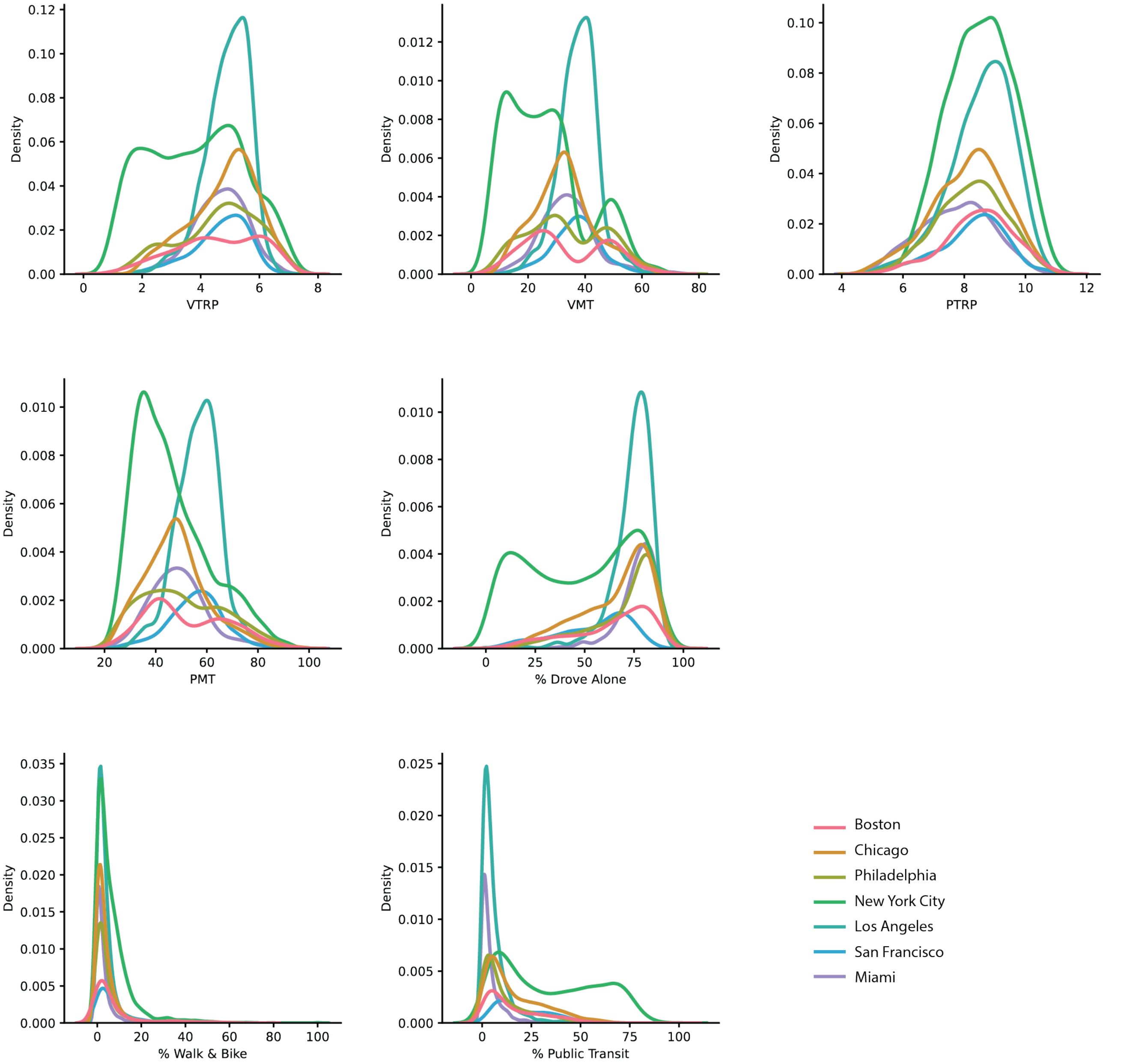

Fig. S5. Distribution of the socioeconomic variables to predict-part A

2. Model Estimation

A. Robustness test with other spatial resolution. To account for the modifiable unit problems (MDU) that often happen in geography studies, we repeat the entire study at both census tract and census block group levels. This section presents the results separately.

A.1. Census Block Group Level Result. All prediction results at the census block group level are shown in Fig. S8 and Table S8.

A.2. Census Tract Level Result. All prediction results at the census tract level are shown in Fig. S9. We also repeat the comparison between POI models and GSV models at the census tract level (Fig. S10)

B. Feature Group Importance Comparison between POI and SVF. To understand the contribution of the POI features and SVF to the overall model prediction, we compute the permutation importance[20] of each feature. The permutation importance p_j for each is defined as:

$$p_j = s - \frac{1}{K} \sum_{k=}^{K} s_{k,j} \qquad [1]$$

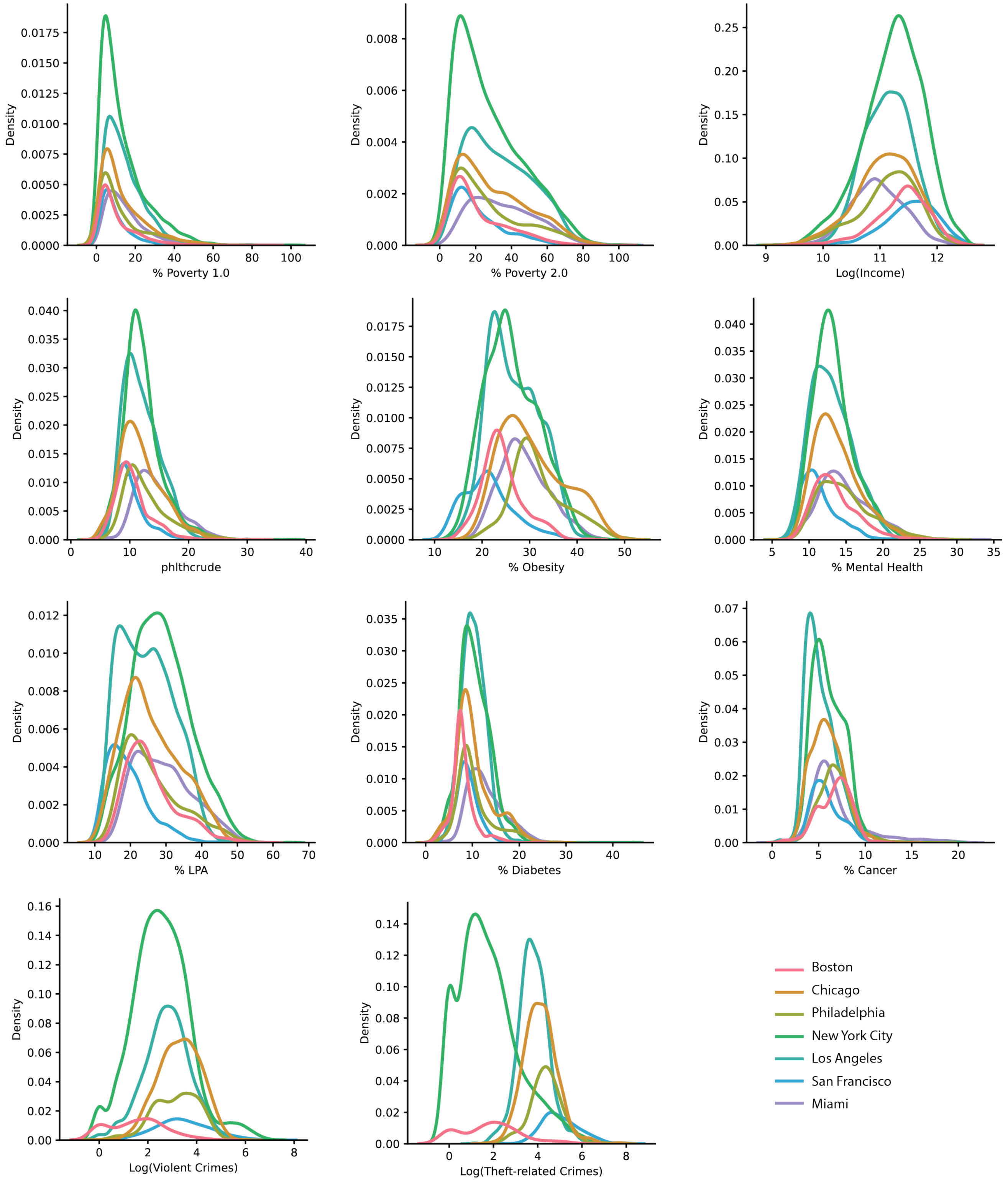

Fig. S6. Distribution of the socioeconomic variables to predict-part B

where s is the total R^2 of our models. We repeat the calculation for $K = 10$ times for each random seed. Each time, we compute the score $s_{k,j}$ of the model on the dataset. The average permutation importance of the model $Y \sim \{GSV\} + \{POI\}$ is shown in Fig. S11.

Table S6. Summary Statistics of All Dependent Variables (CT level)

Topic	Y	count	mean	std	min	max
Crime	Log(Violent Crime)	4605	2.77	1.11	0.00	6.91
	Log(Theft-related Crime)	4605	2.96	1.59	0.00	7.91
Non-vehicle Travel	% Walk+Bike	13104	5.19	8.26	0.00	100.00
	% Public Transit	13104	18.68	20.67	0.00	100.00
Vehicle Travel	% Drove Alone	13104	62.05	23.09	0.00	100.00
	VMT	12608	32.11	12.50	4.66	73.70
	VTRP	12604	4.47	1.34	0.88	7.28
	PTRP	12628	8.36	1.14	4.67	11.26
	PMT	12614	50.39	13.67	21.43	98.97
Health	%Obesity	11450	27.37	6.26	11.90	50.80
	%Diabetes	11450	10.36	3.56	0.70	44.20
	%LPA	11450	26.25	8.10	10.50	63.70
	%Mental Health	11450	13.55	3.16	6.50	31.90
	%Physical Health	11450	12.12	3.52	3.30	37.60
	%Cancer	11450	6.02	1.99	0.60	20.40
Poverty	Log(Median Household Income)	13061	11.19	0.51	9.13	12.43
	%Poverty 2.0	13099	29.15	18.68	0.00	100.00
	%Poverty 2.0	13099	13.28	11.14	0.00	100.00

C. Compare the effects of SVF, POI, and Dynamic Population. To compare the model fit (R^2) among SVF, POI, and dynamic population, we conduct t-test for model results at both census tract (CT) and census block group (CBG) levels S7.

Table S7. Compare model fits among SVF, POI, and dynamic population at census tract (CT) and census block group (CBG) levels

Spatial Unit	$R^2(POI) - R^2(Pop.)$	t-value	p-value	Obs
CT	0.975	2.711	0.007	118
CBG	0.087	3.274	0.001	118
	$R^2(GSV) - R^2(Pop.)$			
CT	0.228	9.581	0.000	118
CBG	0.214	9.200	0.000	118

Table S8. Full Model Results (CBG level)

			SVF		POI	
city	topic	variables	trainR2	testR2	trainR2	testR2
Boston	Crime	Log(Petty Crime)	0.2169	0.1346	0.3154	0.2254
		Log(Violent Crime)	0.2791	0.1885	0.2530	0.1668
	Health	% Cancer Health	0.4871	0.4211	0.4601	0.4352
		% Diabetes	0.2501	0.2401	0.2113	0.2155
		% LPA	0.3986	0.3752	0.2838	0.2769
		% Mental Health	0.3798	0.3095	0.3528	0.3319
		% Obesity	0.2593	0.1419	0.3214	0.3321
		%Physical Health	0.3061	0.2036	0.2741	0.2763
	Non-vehicle Travel	%Public Transit	0.5250	0.5071	0.4565	0.4323
		%Walk	0.6285	0.6170	0.4210	0.4161
	Vehicle Travel	%Drive Alone	0.6731	0.6581	0.5487	0.5301
		PMT	0.7351	0.6131	0.5308	0.5165
		PTRP	0.4981	0.4329	0.3854	0.3657
		VMT	0.7902	0.6817	0.5783	0.5535
		VTRP	0.7915	0.7623	0.5900	0.5805
	Poverty	% Poverty Line (100%)	0.2077	0.1619	0.1677	0.1161
		% Poverty Line (200%)	0.3719	0.3076	0.2463	0.2324
		Log(Income)	0.3763	0.3581	0.2405	0.2400
Chicago	Crime	Log(Petty Crime)	0.3687	0.3296	0.4565	0.4188
		Log(Violent Crime)	0.4573	0.4301	0.3898	0.3576
	Health	% Cancer Health	0.3800	0.3695	0.3162	0.2908
		% Diabetes	0.5183	0.5043	0.4431	0.4446
		% LPA	0.5084	0.4915	0.4981	0.4962
		% Mental Health	0.4549	0.4330	0.4556	0.4430
		% Obesity	0.4572	0.4456	0.3836	0.3742
		% Physical Health	0.5065	0.4866	0.4674	0.4595
	Non-vehicle Travel	% Public Transit	0.5585	0.5565	0.4250	0.4121
		% Walk	0.3954	0.3677	0.2825	0.2395
	Vehicle Travel	% Drive Alone	0.6303	0.6200	0.4436	0.4218
		PMT	0.5995	0.5923	0.4825	0.4621
		PTRP	0.4682	0.4650	0.3008	0.2978
		VMT	0.6471	0.6393	0.5367	0.5183
		VTRP	0.6667	0.6478	0.4874	0.4833
	Poverty	% Poverty Line (100%)	0.2006	0.1801	0.1629	0.1398
		% Poverty Line (200%)	0.3972	0.3895	0.3744	0.3726
		Log(Income)	0.4337	0.4358	0.3867	0.3803
Los Angeles	Crime	Log(Petty Crime)	0.1339	0.1074	0.3220	0.3049
		Log(Violent Crime)	0.3527	0.3243	0.3373	0.3060
	Health	% Cancer Health	0.4068	0.3703	0.3742	0.3598
		% Diabetes	0.3969	0.3856	0.3842	0.3738
		% LPA	0.5917	0.5786	0.5239	0.5186
		% Mental Health	0.5343	0.5201	0.3809	0.3721
		% Obesity	0.4640	0.4638	0.4254	0.4170
		% Physical Health	0.5241	0.5130	0.4317	0.4219
	Non-vehicle Travel	% Public Transit	0.3332	0.2880	0.2446	0.2351
		% Walk	0.1536	0.1566	0.1384	0.1356
	Vehicle Travel	% Drive Alone	0.2514	0.2220	0.1750	-1.5694
		PMT	0.4529	0.4527	0.2595	0.2505
		PTRP	0.4387	0.4367	0.2546	0.2466
		VMT	0.4885	0.4682	0.2921	0.2848
		VTRP	0.5314	0.5404	0.2864	0.2841

	Poverty	% Poverty Line (100%)	0.1261	0.1169	0.0814	0.0668
		% Poverty Line (200%)	0.4444	0.4356	0.3411	0.3406
		Log(Income)	0.4573	0.4518	0.3603	0.3528
Miami	Health	% Cancer Health	0.1991	0.1566	0.2459	0.2312
		% Diabetes	0.4527	0.4342	0.3237	0.3055
		% LPA	0.5686	0.5528	0.4544	0.4279
		% Mental Health	0.3312	0.3107	0.2970	0.2570
		% Obesity	0.4148	0.3874	0.3874	0.3484
		% Physical Health	0.4602	0.4426	0.3268	0.3042
	Non-vehicle Travel	% Public Transit	0.2262	0.2090	0.1235	0.0986
		% Walk	0.2744	0.2727	0.1541	0.1255
	Vehicle Travel	% Drive Alone	0.1921	0.1726	0.1077	0.0810
		PMT	0.3625	0.3560	0.2589	0.2438
		PTRP	0.3037	0.2738	0.2624	0.2235
		VMT	0.3537	0.3290	0.2467	0.2122
		VTRP	0.3868	0.3678	0.2680	0.2308
	Poverty	% Poverty Line (200%)	0.3809	0.3566	0.2690	0.2528
		Log(Income)	0.4215	0.3993	0.2502	0.2166
New York	Crime	Log(Petty Crime)	0.1998	0.1964	0.2913	0.2769
		Log(Violent Crime)	0.2301	0.2228	0.2134	0.2045
	Health	% Cancer Health	0.4886	0.4783	0.4279	0.4134
		% Diabetes	0.4585	0.3047	0.3062	0.2997
		% LPA	0.5457	0.4995	0.3510	0.3419
		% Mental Health	0.4069	0.3034	0.3055	0.2920
		% Obesity	0.3923	0.2478	0.3095	0.2964
		% Physical Health	0.4263	0.3152	0.2774	0.2661
	Non-vehicle Travel	% Public Transit	0.7332	0.7265	0.5670	0.5619
		% Walk	0.4117	0.4177	0.2486	0.2449
	Vehicle Travel	% Drive Alone	0.8121	0.8071	0.5929	0.5842
		PMT	0.7423	0.7383	0.5455	0.5407
		PTRP	0.4318	0.4261	0.2591	0.2573
		VMT	0.8122	0.8049	0.6185	0.6010
		VTRP	0.8544	0.8532	0.6229	0.6215
	Poverty	% Poverty Line (100%)	0.1797	0.1643	0.1249	0.1196
		% Poverty Line (200%)	0.4084	0.3909	0.2907	0.2837
		Log(Income)	0.4328	0.4310	0.2810	0.2804
Philadelphia	Crime	Log(Petty Crime)	0.3602	0.2872	0.5432	0.4601
		Log(Violent Crime)	0.4508	0.4352	0.2846	0.1995
	Health	% Cancer Health	0.4080	0.3892	0.3379	0.2943
		% Diabetes	0.5161	0.5141	0.4014	0.3767
		% LPA	0.6815	0.6753	0.4849	0.4665
		% Mental Health	0.6702	0.6643	0.4650	0.4548
		% Obesity	0.4609	0.4429	0.3368	0.3124
		% Physical Health	0.6287	0.6215	0.4457	0.4289
	Non-vehicle Travel	% Public Transit	0.5249	0.5153	0.3880	0.3869
		% Walk	0.5017	0.4787	0.2921	0.2576
	Vehicle Travel	% Drive Alone	0.6038	0.5861	0.4173	0.3991
		PMT	0.7549	0.7503	0.5603	0.5484
		PTRP	0.4732	0.4768	0.2849	0.2593
		VMT	0.7822	0.7812	0.5932	0.5792
		VTRP	0.7670	0.7722	0.5665	0.5632
	Poverty	% Poverty Line (100%)	0.2904	0.2867	0.1776	0.1832
		% Poverty Line (200%)	0.5397	0.5347	0.3555	0.3434
		Log(Income)	0.5311	0.5384	0.3687	0.3637
San Francisco	Crime	Log(Petty Crime)	0.5286	0.5042	0.5600	0.5373

	Log(Violent Crime)	0.5348	0.4826	0.4918	0.4632
Health	% Cancer Health	0.4739	0.4596	0.3457	0.3173
	% Diabetes	0.4104	0.3692	0.2710	0.2378
	% LPA	0.5317	0.4856	0.3633	0.3423
	% Mental Health	0.4353	0.3869	0.2809	0.2445
	% Obesity	0.5547	0.5125	0.2890	0.2647
	% Physical Health	0.4619	0.4146	0.2961	0.2668
Non-vehicle Travel	% Public Transit	0.5767	0.5654	0.4182	0.4050
	% Walk	0.6113	0.5605	0.4121	0.3624
Vehicle Travel	% Drive Alone	0.6949	0.6837	0.4655	0.4410
	PMT	0.5863	0.5517	0.3639	0.3170
	PTRP	0.5299	0.5365	0.3574	0.3474
	VMT	0.6260	0.6070	0.3885	0.3507
	VTRP	0.6714	0.6505	0.4084	0.3766
Poverty	% Poverty Line (100%)	0.1366	0.1226	0.1220	0.0800
	% Poverty Line (200%)	0.3355	0.3059	0.2200	0.1878
	Log(Income)	0.3633	0.3654	0.2586	0.2028

Table S9. Full Model Results (CT level)

city	topic	variables	GSV trainR2	testR2	POI testR2	trainR2
Boston	Crime	Log(Violent Crime)	0.2602	0.1394	0.1444	0.2444
		Log(Petty Crime)	0.2104	0.1055	0.2571	0.3702
	Health	% Cancer Health	0.5265	0.4806	0.4853	0.5507
		% Diabetes	0.3563	0.2941	0.3042	0.3588
		% LPA	0.5048	0.4173	0.3795	0.4463
		% Mental Health	0.4688	0.4095	0.4031	0.4625
		% Obesity	0.4075	0.3272	0.4547	0.5017
		% Physical Health	0.4373	0.3667	0.3685	0.4234
	Non-vehicle Travel	%Public Transit	0.6462	0.6071	0.6139	0.6502
		%Walk	0.7729	0.7459	0.5241	0.5902
	Poverty	Log(Income)	0.5430	0.4909	0.3819	0.4478
		% Poverty Line (100%)	0.4629	0.4085	0.3445	0.4081
		% Poverty Line (200%)	0.5516	0.4969	0.4183	0.4684
	Vehicle Travel	% Drive Alone	0.8081	0.7767	0.7361	0.7594
		PMT	0.7945	0.7804	0.6448	0.6703
		PTRP	0.5562	0.5057	0.4552	0.4919
		VMT	0.8460	0.8258	0.6749	0.7163
		VTRP	0.8349	0.8145	0.7026	0.7220
Chicago	Crime	Log(Violent Crime)	0.4450	0.3582	0.4756	0.5547
		Log(Petty Crime)	0.2845	0.1571	0.4891	0.5774
	Health	% Cancer Health	0.4085	0.3713	0.3527	0.3888
		% Diabetes	0.6195	0.5935	0.5981	0.6174
		% LPA	0.5988	0.5759	0.6370	0.6563
		% Mental Health	0.5153	0.4897	0.5658	0.5836
		% Obesity	0.5578	0.5263	0.5702	0.5822
		% Physical Health	0.5920	0.5703	0.5886	0.6088
	Non-vehicle Travel	%Public Transit	0.7096	0.6822	0.6255	0.6509
		%Walk	0.4651	0.4217	0.3306	0.4011
	Poverty	Log(Income)	0.5798	0.5638	0.5891	0.6012
		% Poverty Line (100%)	0.5121	0.4942	0.5104	0.5240
		% Poverty Line (200%)	0.5610	0.5440	0.6082	0.6213
	Vehicle Travel	% Drive Alone	0.7966	0.7718	0.6236	0.6686

		PMT	0.6553	0.6385	0.5408	0.5682
		PTRP	0.5166	0.4840	0.3522	0.4042
		VMT	0.7006	0.6627	0.6019	0.6278
		VTRP	0.7070	0.6847	0.5956	0.6122
Los Angeles	Crime	Log(Violent Crime)	0.4693	0.4393	0.4756	0.4907
		Log(Petty Crime)	0.1985	0.1062	0.4676	0.4754
	Health	% Cancer Health	0.4410	0.4428	0.3923	0.4221
		% Diabetes	0.4444	0.4279	0.4194	0.4591
		% LPA	0.6618	0.6561	0.6512	0.6518
		% Mental Health	0.5692	0.5626	0.4422	0.4506
		% Obesity	0.5418	0.5207	0.4992	0.5111
		% Physical Health	0.5876	0.5602	0.4579	0.5197
	Non-vehicle Travel	%Public Transit	0.5219	0.4877	0.3169	0.3592
		%Walk	0.1890	0.1715	0.0223	0.0803
	Poverty	Log(Income)	0.5995	0.5684	0.4954	0.5243
		% Poverty Line (100%)	0.4390	0.3896	0.2947	0.3276
		% Poverty Line (200%)	0.5939	0.5642	0.4722	0.5018
	Vehicle Travel	% Drive Alone	0.4559	0.4567	0.2366	0.2529
		PMT	0.5050	0.4953	-0.9921	0.3332
		PTRP	0.4983	0.4710	0.3040	0.3406
		VMT	0.5424	0.5325	-2.4079	0.3641
		VTRP	0.5891	0.5549	0.0614	0.3635
Miami	Health	% Cancer Health	0.2449	0.2118	0.3492	0.3582
		% Diabetes	0.4472	0.4237	0.3369	0.3705
		% LPA	0.6015	0.5897	0.5292	0.5574
		% Mental Health	0.3510	0.3114	0.3145	0.3830
		% Obesity	0.4388	0.4057	0.4395	0.4825
		% Physical Health	0.4688	0.4476	0.3497	0.3930
	Non-vehicle Travel	%Public Transit	0.3959	0.3839	0.2264	0.2652
		%Walk	0.4408	0.3971	0.2208	0.3030
	Poverty	Log(Income)	0.5180	0.5081	0.3535	0.3929
		% Poverty Line (100%)	0.3925	0.3371	0.2976	0.3618
		% Poverty Line (200%)	0.5053	0.4825	0.4015	0.4365
	Vehicle Travel	% Drive Alone	0.3762	0.3747	0.1864	0.2385
		PMT	0.4551	0.3974	0.3018	0.3495
		PTRP	0.3971	0.3276	0.3080	0.3490
		VMT	0.4189	0.3952	0.2928	0.3286
		VTRP	0.4399	0.4154	0.3128	0.3355
New York	Crime	Log(Violent Crime)	0.3267	0.2942	0.3295	0.3496
		Log(Petty Crime)	0.3364	0.3387	0.3923	0.3923
	Health	% Cancer Health	0.4562	0.4135	0.0362	0.4679
		% Diabetes	0.4590	0.4484	0.3401	0.3593
		% LPA	0.5385	0.5126	0.4046	0.4293
		% Mental Health	0.3855	0.3460	0.3261	0.3566
		% Obesity	0.4250	0.3900	0.3504	0.3819
		% Physical Health	0.4006	0.3789	0.2996	0.3189
	Non-vehicle Travel	%Public Transit	0.8212	0.8193	0.6998	0.7084
		%Walk	0.5317	0.5014	0.2909	0.3114
	Poverty	Log(Income)	0.5221	0.5082	0.4322	0.4496
		% Poverty Line (100%)	0.4198	0.3967	0.3559	0.3644
		% Poverty Line (200%)	0.4978	0.4790	0.4493	0.4603
	Vehicle Travel	% Drive Alone	0.8844	0.8789	0.7095	0.7172
		PMT	0.7509	0.7437	0.5444	0.6085
		PTRP	0.4173	0.3958	0.2641	0.2818
		VMT	0.8199	0.8166	0.6502	0.6785

		VTRP	0.8544	0.8452	0.7006	0.7127
Philadelphia	Crime	Log(Violent Crime)	0.7000	0.6392	0.4307	0.5183
		Log(Petty Crime)	0.4733	0.4091	0.5787	0.6476
	Health	% Cancer Health	0.3812	0.3659	0.3590	0.3962
		% Diabetes	0.5245	0.4622	0.4469	0.4862
		% LPA	0.6825	0.6430	0.5522	0.5762
		% Mental Health	0.6499	0.6203	0.5162	0.5487
		% Obesity	0.4661	0.3954	0.3738	0.4231
		% Physical Health	0.6319	0.5767	0.4975	0.5284
	Non-vehicle Travel	%Public Transit	0.6766	0.6829	0.5700	0.5665
		%Walk	0.6619	0.6256	0.4270	0.4936
	Poverty	Log(Income)	0.6303	0.5970	0.5235	0.5451
		% Poverty Line (100%)	0.5874	0.5490	0.4098	0.4634
		% Poverty Line (200%)	0.6585	0.6248	0.5042	0.5338
	Vehicle Travel	% Drive Alone	0.7727	0.7818	0.6459	0.6458
		PMT	0.7412	0.7394	0.6267	0.6202
		PTRP	0.4743	0.4478	0.3279	0.3628
		VMT	0.7815	0.7586	0.6482	0.6635
		VTRP	0.7611	0.7689	0.6507	0.6574
San Francisco	Crime	Log(Violent Crime)	0.6414	0.4717	0.5579	0.7200
		Log(Petty Crime)	0.6141	0.4457	0.5609	0.7138
	Health	% Cancer Health	0.5117	0.4404	-0.1123	0.3747
		% Diabetes	0.4009	0.3418	0.2765	0.3493
		% LPA	0.5413	0.4790	0.3931	0.4738
		% Mental Health	0.4776	0.4014	0.1949	0.3519
		% Obesity	0.5906	0.5402	0.2431	0.3563
		% Physical Health	0.4857	0.4167	0.3052	0.3835
	Non-vehicle Travel	%Public Transit	0.7260	0.6827	0.5257	0.5990
		%Walk	0.7826	0.7140	0.5038	0.6095
	Poverty	Log(Income)	0.4720	0.3907	0.2472	0.3675
		% Poverty Line (100%)	0.3729	0.3083	0.1836	0.2661
		% Poverty Line (200%)	0.4660	0.3953	0.2473	0.3310
	Vehicle Travel	% Drive Alone	0.8336	0.8267	0.5410	0.6401
		PMT	0.6210	0.6125	0.3687	0.4205
		PTRP	0.5669	0.5103	0.4094	0.4540
		VMT	0.6704	0.6470	0.3146	0.4629
		VTRP	0.7043	0.6491	0.4503	0.5003

3. Software used

All studies in this research are conducted with Python 3.8. Part of the visualizations is done through QGIS.

4. Code Availability

The analysis was conducted using Python. Code to reproduce the main results in the figures from the aggregated data (data link included) is publicly available on a GitHub repository (https://github.com/brookefzy/urban-visual-intelligence).

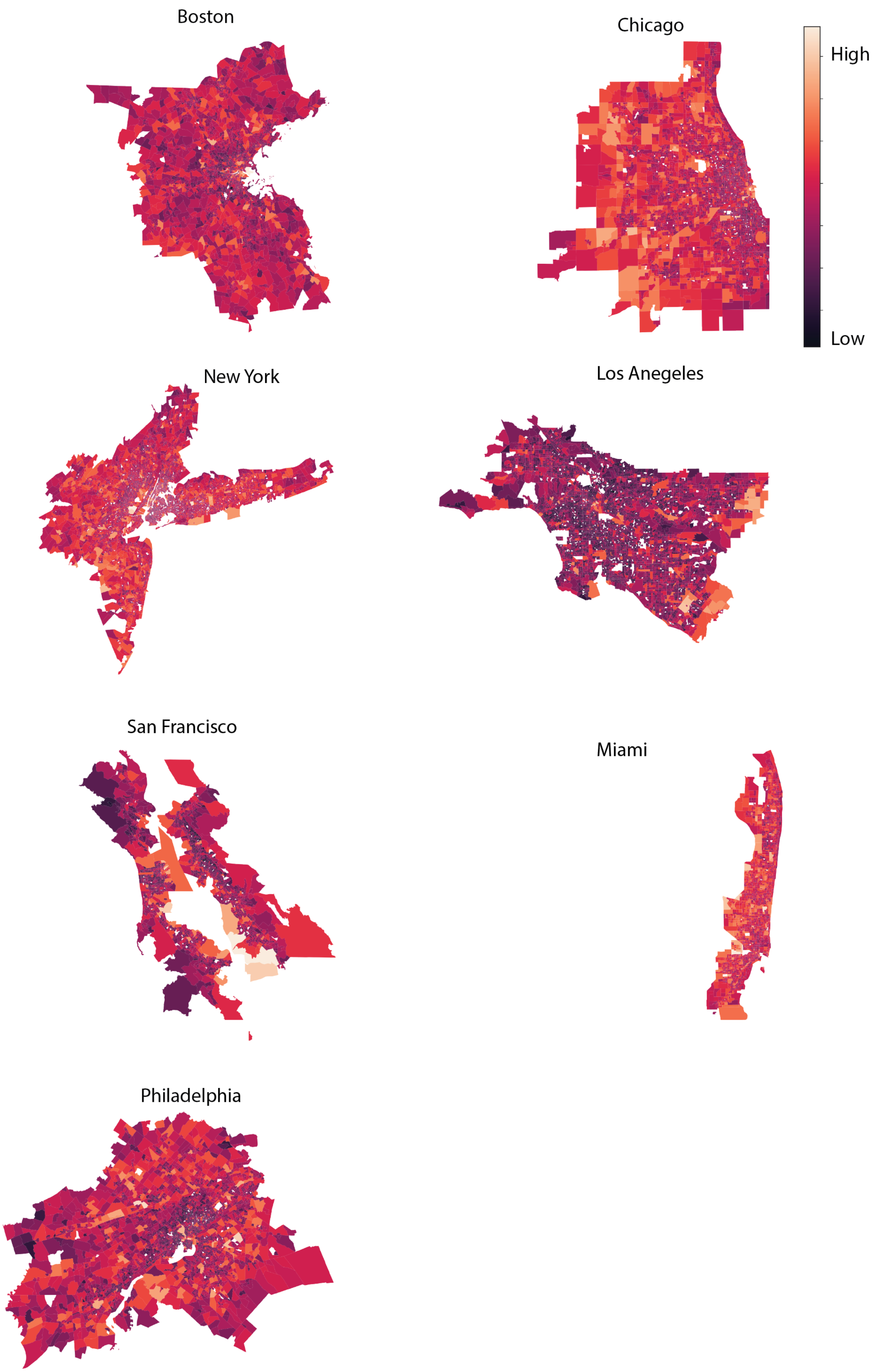

Fig. S7. Spatial distribution of average summer visiting volume on a weekday

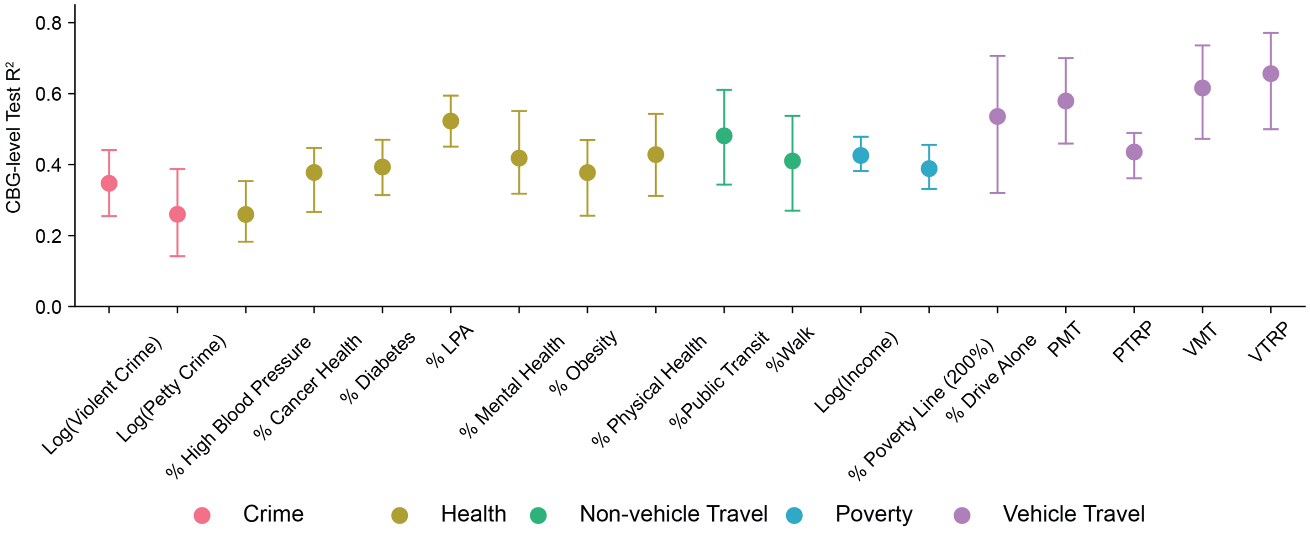

Fig. S8. All census block group (CBG)-level models in detail

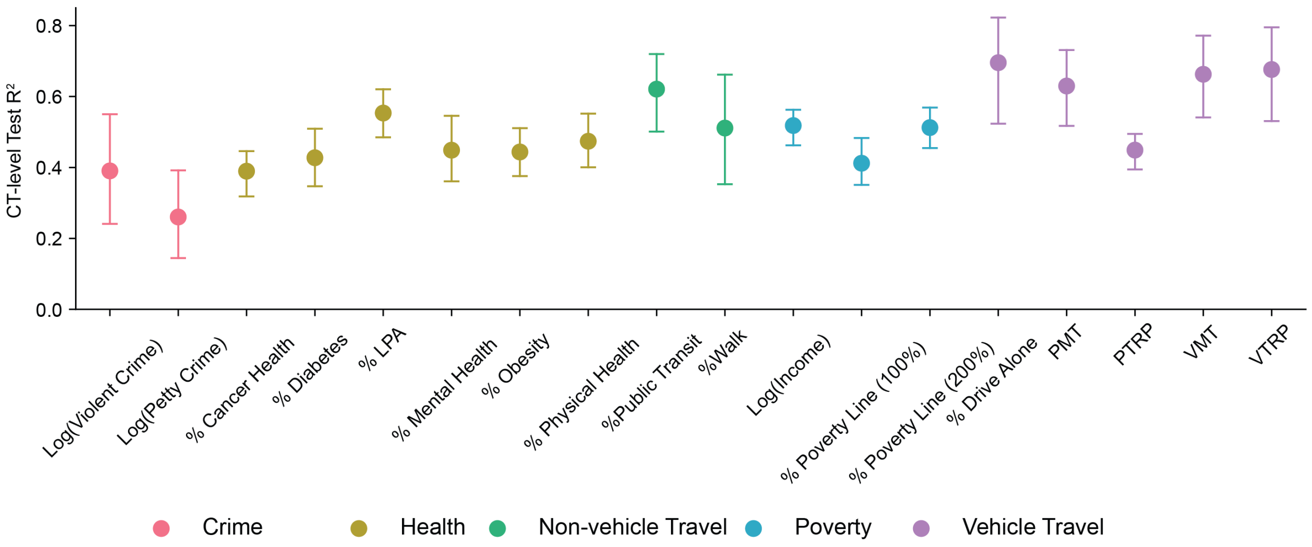

Fig. S9. All census tract (CT)-level models in detail

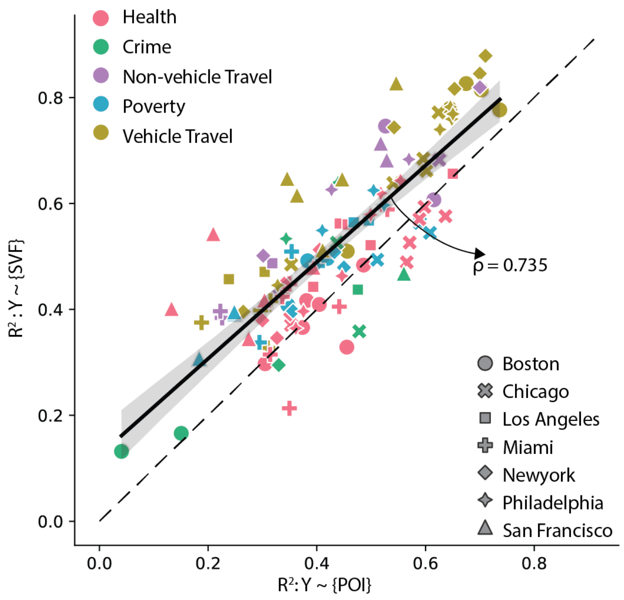

Fig. S10. Compare the POI models with SVF models at the census track (CT) level. The shading indicates 95% confidence interval.

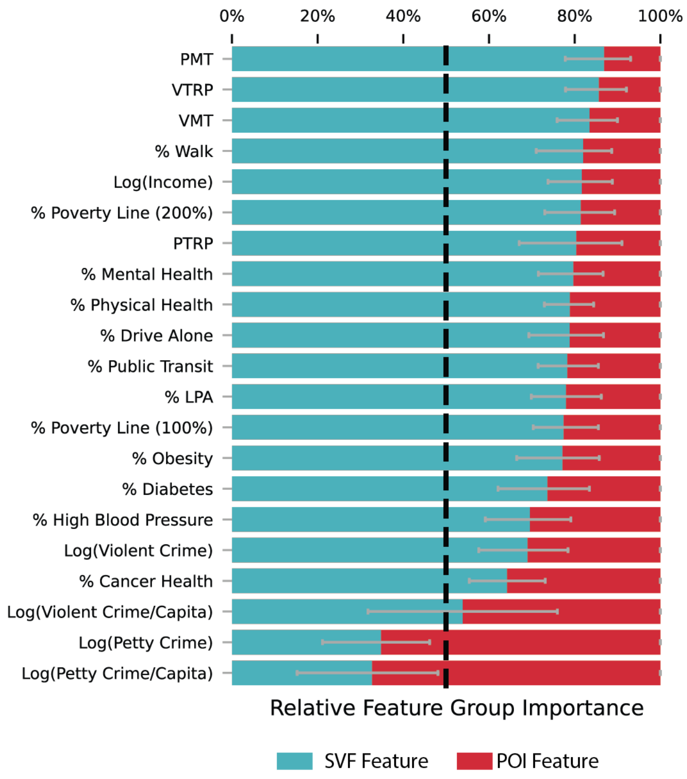

Fig. S11. Relative permutation importance of SVF and POI features

References

[1] B Zhou, et al., Semantic understanding of scenes through the ade20k dataset. *Int. J. on Comput. Vis.* (2018).
[2] F Zhang, D Zhang, Y Liu, H Lin, Representing place locales using scene elements. *Comput. Environ. Urban Syst.* **71**, 153–164 (2018).
[3] A Forsyth, M Southworth, Cities afoot—pedestrians, walkability and urban design (2008).
[4] Z Guo, BP Loo, Pedestrian environment and route choice: evidence from new york city and hong kong. *J. transport geography* **28**, 124–136 (2013).
[5] A Sevtsuk, R Kalvo, Predicting pedestrian flow along city streets: A comparison of route choice estimation approaches in downtown san francisco. *Int. J. Sustain. Transp.* pp. 1–15 (2020).
[6] J Jacobs, *The death and life of great American cities*. (Vintage), (2016).
[7] MA Andresen, The ambient population and crime analysis. *The Prof. Geogr.* **63**, 193–212 (2011).
[8] Safegraph, Safegraph places data (https://docs.safegraph.com/docs/places) (year?) Accessed: 2020.
[9] N Park, Recreation, Park area (https://www.arcgis.com/home/item.html?id=578968f975774d3fab79fe56c8c90941) (2020) Accessed: 2020.
[10] Center for Transit-Oriented Development, National tod database (https://toddata.cnt.org/) (2011) Accessed: 2020.
[11] Reference USA, Reference usa historical dataset (https://dataverse.harvard.edu/dataset.xhtml?persistentId=doi:10.7910/DVN/GW2P3G) (2019) Accessed: 2020.
[12] Safegraph, Weekly patterns (https://docs.safegraph.com/docs/weekly-patterns) (2019) Accessed: 2020.
[13] New York City Police Department, City wide crime statistic incident level data (https://www.nyc.gov/site/nypd/stats/crime-statistics/citywide-crime-stats.page) (2019) Accessed: 2021.
[14] Boston Police Department, Analyze boston crime incident reports (https://data.boston.gov/dataset/crime-incident-reports-august-2015-to-date-source-new-system) (2019) Accessed: 2021.
[15] City of Philadelphia, Opendataphilly crime incidents (https://www.opendataphilly.org/dataset/crime-incidents) (2019) Accessed: 2021.
[16] City of Cambridge Police Department, Cambrdige open data crime reports (https://data.cambridgema.gov/Public-Safety/Crime-Reports/xuad-73uj) (2019) Accessed: 2021.
[17] City of Los Angeles, Crime data from 2010 to 2019 (https://data.lacity.org/Public-Safety/Crime-Data-from-2010-to-2019/63jg-8b9z) (2019) Accessed: 2021.
[18] City and County of San Francisco, Police department incident reports (https://data.sfgov.org/Public-Safety/Police-Department-Incident-Reports-Historical-2003/tmnf-yvry) (2019) Accessed: 2021.
[19] City of Chicago, Crimes - 2001 to present (https://data.cityofchicago.org/Public-Safety/Crimes-2019/w98m-zvie) (2019) Accessed: 2021.
[20] A Fisher, C Rudin, F Dominici, All models are wrong, but many are useful: Learning a variable's importance by studying an entire class of prediction models simultaneously. *J. Mach. Learn. Res.* **20**, 1–81 (2019).
